# An NMR-Based Model to Investigate the Metabolic Phenoreversion of COVID-19 Patients throughout a Longitudinal Study

**DOI:** 10.3390/metabo12121206

**Published:** 2022-12-01

**Authors:** Rubén Gil-Redondo, Ricardo Conde, Maider Bizkarguenaga, Chiara Bruzzone, Ana Laín, Beatriz González-Valle, Milagros Iriberri, Carlos Ramos-Acosta, Eduardo Anguita, Juan Ignacio Arriaga Lariz, Pedro Pablo España Yandiola, Miguel Ángel Moran, Mario Ernesto Jiménez-Mercado, Leire Egia-Mendikute, María Luisa Seco, Hartmut Schäfer, Claire Cannet, Manfred Spraul, Asís Palazón, Nieves Embade, Shelly C. Lu, Julien Wist, Jeremy K. Nicholson, José M. Mato, Oscar Millet

**Affiliations:** 1 Precision Medicine and Metabolism Laboratory, CIC bioGUNE, Basque Research and Technology Alliance (BRTA), 48160 Derio, Spain; 2 Pneumology Department, Hospital Universitario de Cruces, 48903 Barakaldo, Spain; 3 Department of Medicine, Faculty of Medicine, UCM, Hematology Department, IML, IdISSC, Hospital Clínico San Carlos, 28040 Madrid, Spain; 4 Pneumology Department, Hospital Universitario de Basurto, 48013 Bilbao, Spain; 5 Pneumology Department, Hospital Universitario de Galdakao-Usansolo, 48960 Galdakao, Spain; 6 Bioaraba Health Research Institute, 01009 Vitoria-Gasteiz, Spain; 7 Internal Medicine Department, Infectious Diseases Section, Osakidetza Basque Health Service, Araba University Hospital, 01009 Vitoria-Gasteiz, Spain; 8 Emergency Department, Osakidetza Basque Health Service, Araba University Hospital, 01009 Vitoria-Gasteiz, Spain; 9 Cancer Immunology and Immunotherapy Lab, CIC bioGUNE, Basque Research and Technology Alliance (BRTA), 48160 Derio, Spain; 10 OSARTEN Kooperatiba Elkartea, 20500 Arrasate-Mondragón, Spain; 11 Bruker Biospin GmbH, Silberstreifen, 76287 Rheinstetten, Germany; 12 Ikerbasque, Basque Foundation for Science, 48015 Derio, Spain; 13 Karsh Division of Gastroenterology and Hepatology, Cedars-Sinai Medical Center, Los Angeles, CA 90048, USA; 14 Australian National Phenome Center, and Center for Computational and Systems Medicine, Health Futures Institute, Murdoch University, Harry Perkins Building, Perth, WA 6150, Australia; 15 Chemistry Department, Universidad del Valle, Cali 76001, Colombia; 16 Medical School, Faculty of Health and Medical Sciences, University of Western Australia, Department of Endocrinology and Diabetes, Fiona Stanley Hospital, Perth, WA 6150, Australia; 17 Institute of Global Health Innovation, Faculty of Medicine, Imperial College London, Level 1, Faculty Building, South Kensington Campus, London SW7 2NA, UK; 18 CIBERehd, Instituto de Salud Carlos III, 28029 Madrid, Spain

**Keywords:** COVID-19, atherosclerotic risk, metabolomics, lipidomics, inflammation, long COVID

## Abstract

After SARS-CoV-2 infection, the molecular phenoreversion of the immunological response and its associated metabolic dysregulation are required for a full recovery of the patient. This process is patient-dependent due to the manifold possibilities induced by virus severity, its phylogenic evolution and the vaccination status of the population. We have here investigated the natural history of COVID-19 disease at the molecular level, characterizing the metabolic and immunological phenoreversion over time in large cohorts of hospitalized severe patients (n = 886) and non-hospitalized recovered patients that self-reported having passed the disease (n = 513). Non-hospitalized recovered patients do not show any metabolic fingerprint associated with the disease or immune alterations. Acute patients are characterized by the metabolic and lipidomic dysregulation that accompanies the exacerbated immunological response, resulting in a slow recovery time with a maximum probability of around 62 days. As a manifestation of the heterogeneity in the metabolic phenoreversion, age and severity become factors that modulate their normalization time which, in turn, correlates with changes in the atherogenesis-associated chemokine MCP-1. Our results are consistent with a model where the slow metabolic normalization in acute patients results in enhanced atherosclerotic risk, in line with the recent observation of an elevated number of cardiovascular episodes found in post-COVID-19 cohorts.

## 1. Introduction

Acute COVID-19 patients have been associated with a characteristic metabotype [1,2,3,4,5], reflecting the systemic character of the disease. This metabolic dysregulation includes central metabolites such as glucose, amino acids including arginine, glutamate and the tryptophan/kynurenine pathway, porphyrins, lipids and the overall serum lipoprotein composition [5,6,7]. Genetic alterations have also been associated with severe phenotypes of COVID-19 [8,9] and, importantly, the characteristic metabotype associated with SARS-CoV-2 infection qualitatively correlates with the inflammatory response observed in hospitalized patients [10]. Thus, the restoration of the immunological response and the normalization of the metabolic signature (phenoreversion) are both required for full recovery of the disease [11,12] but the timescale of this normalization has not yet been studied in-depth due to the limitations in the cohort size and the time frame of the existing longitudinal studies [13].

Aside from the more-characterized hospitalized patients, the bulk of SARS-CoV-2 infections corresponds to mild or asymptomatic patients that undergo the disease without a significant immune stress response [14]. These individuals are largely unnoticed by the healthcare systems, and it is often difficult to obtain samples with the associated COVID-19 status from these cohorts. Consequently, the putative molecular fingerprint from mild or asymptomatic patients has not been characterized with the same degree as acute patients.

The characteristic metabotype observed in acute COVID-19 patients suggests that metabolomics may be a useful tool to investigate the natural history of the disease (i.e., the time course of the disease towards recovery) [15]. Yet, during the pandemic, the virus has evolved extensively both in genotype and virulence. On the other hand, the host has also changed because of global vaccination. Thus, the natural history becomes manifold, with several factors including severity, the lineage of the virus and the vaccination/medication status of the patient largely modulating disease recovery at the molecular level.

Here, we have used NMR-based metabolomics and immunological assays to investigate the natural history of COVID-19 disease at the metabolic level. Most metabolomic studies either use gas chromatography (GC) or liquid chromatography (LC) coupled to mass spectrometry (MS) or NMR spectroscopy [16,17]. LC-MS and GC-MS show high sensitivity, which enables monitoring the entire metabolism, but at the cost of limited reproducibility since they may require sample derivatization and quantification relies on the use of standards. Instead, NMR spectroscopy is highly complementary since it is fully quantitative, requires no derivatization and it is very reproducible, but with low sensitivity, which limits the accessible metabolome that can be investigated by this technique.

We have considered two different evolution scenarios of the disease: hospitalized patients that evolve from an acute symptomatic phase towards full recovery and non-hospitalized patients who self-reported having passed the disease and that already donate samples in the recovery period (>7 days after the onset of the disease). Both possible disease scenarios correspond to extremely opposite evolutions of the disease and they inflict a very different effect on the serum metabolism, which in turn, is largely proportional to the immune response [10]. Age at the disease onset is a risk factor for metabolic recovery after COVID-19 infection while a post-vaccination status of the donor results in a negligible effect in our metabolic model of the serum metabolism.

## 2. Materials and Methods

### 2.1. Patient Recruitment and Sample Collection

COVID-19 cohorts were recruited from patients from Araba University Hospital, Basurto University Hospital, Cruces University Hospital, Galdakao Hospital (Basque Country), or San Carlos Clinical Hospital (Madrid). All enrolled patients tested positive for SARS-CoV-2 infection from RT−PCR on nasopharyngeal swab samples during their hospitalization. Control samples were acquired either pre-COVID-19 pandemic or during 2021 by Osarten Kooperativa Elkartea from employees of the Mondragon Cooperative (Basque Country) in overnight fasting conditions and during the annual medical test. Both cohorts were provided by the Basque Biobank for Research (BIOEF). Infection and vaccination statuses for 2021 donors were ascertained using a questionnaire.

### 2.2. Sample Preparation, Instrumentation, and Sample Acquisition with IVDr NMR Metabolomics

Serum samples were handled under the same standard operating procedures and stored at −80 °C until analysis. They were prepared both manually and automatically. For manual preparation, serum was mixed with serum buffer (75 mM Na_2_HPO_4_, 2 mM NaN_3_, 4.6 mM sodium trimethylsilyl propionate-[2,2,3,3-^2^H_4_] (TSP) in 10% D_2_O, pH 7.4  ±  0.1) in a 1:1 (*v*/*v*) ratio for a final volume of 600 µL into the 5 mm NMR tube. For automatic preparation, a SamplePro Tube (Bruker BioSpin, Germany) robot system for liquid handling with integrated temperature control was used. All samples were automatically prepared with serum buffer at a 1:1 (*v*/*v*) ratio, at a final volume of 600 μL in 5 mm NMR tubes. NMR measurements were done in a 600 MHz IVDr (Bruker BioSpin, Silberstreifen, Germany) with a tempered SampleJet automatic sample changer mounted on it and a double resonance broadband probe (BBI) probe head with a z gradient coil and BOSS-III shim system. NMR sample tubes were stored inside the SampleJet at 5 °C until measurement. Every morning the spectrometer was calibrated with three different samples: methanol, QuantRef and sucrose to check the temperature (310 K), the quantification performance and optimal shimming, respectively, following strict standard operation procedures, as previously described [12]. Three different ^1^H NMR experiments were recorded in all samples: a standard one-dimensional (1D) ^1^H NOESY spectrum (noesygppr1d) with water presaturation, a 1D ^1^H Carr–Purcell–Meiboom–Gill (CPMG) experiment (cpmgpr1d) implementing a T_2_ filter to suppress the broad signals of proteins and other macromolecules, and a two-dimensional *J*-resolved experiment (jresgpprqf).

### 2.3. NMR Quantifications

Absolute quantifications from ^1^H-NMR spectra were performed with Bruker IVDr software: B.I.Quant-PS 2.0.0 to quantify 41 serum metabolites (mmol/L units) and B.I.LISA (Lipoprotein Subclass Analysis) PL-5009-01/001 to quantify 112 serum lipoprotein classes and subclasses (mg/dL units except for particle numbers that are expressed as nmol/L and ratios which are dimensionless). Inflammation biomarkers (GlycA, GlycB and SPC) were directly estimated from CPMG spectra, which were segmented into consecutive buckets (bins) of fixed 0.03 ppm spectral width in the range between 0.5 and 9.5 ppm. Each bin was represented as the summed intensity of their internal spectral points and normalized by the total spectrum intensity. The region between 4.7 and 5.00 (residual water) was excluded. GlycA, GlycB and SPC values were taken from bins centered at 2.06, 2.09 and 3.23, respectively.

### 2.4. Cytokine and Chemokine Quantification

The levels of the indicated cytokines and chemokines were measured in serum samples by flow cytometry, using the LEGENDplexTM COVID-19 Cytokine Storm Panel 1 (13-Plex) (BioLegend, San Diego, CA, USA) following the manufacturer’s instructions. A minimum of 600 events for each address were acquired using a FACSymphony flow cytometer (BD Biosciences, San Jose, CA, USA) and the mean fluorescence intensities (MFI) were obtained. Results were analyzed using The LEGENDplex™ Data Analysis Software Suite (BioLegend).

### 2.5. Filtering of Samples

All samples were analyzed with DBSCAN (Density-based spatial clustering of applications with noise) in order to detect and remove extreme individuals (groups with low density or isolated samples). Metabolites, lipoproteins, inflammation biomarkers and calculated parameters were used as input variables after centering and standardization. The used algorithm was implemented in dbscan R package (version 1.1-8). The number of minimum points per region (minPts) was set to 5. After visualization of all 5-NN (nearest neighbors) multivariate Euclidean distances, a value of 20 was selected for the eps parameter. Those samples without assigned clusters (isolated) were discarded.

### 2.6. Nomenclature for Days since COVID

For non-hospitalized recovered (NHR) individuals the number of days since COVID-19 is simply calculated as the difference between the collection date and the COVID-19 reported date. For COVID-19 hospitalized patients, the first collected sample is considered time zero (t = 0) if it was collected up to 7 days after hospitalization. In case of follow-up, the time for the rest of the collected samples for the same person is calculated taking t = 0 as reference. If there is no t = 0 then the number of days since COVID-19 is calculated as the difference between the collection date and the hospitalization date. COVID-19 samples with t = 0 are considered in the acute phase.

### 2.7. Statistical Analysis

A comparison between HC cohort (controls) and AC (0 and 1) samples at t = 0 (cases) was performed through multivariable linear models adjusted by gender and age. For each variable, obtained *p*-values were corrected for multiple testing using the Benjamini and Hochberg method (FDR—False Discovery Rate) to control for type I errors. The effect size of COVID-19 against the control was expressed as standard deviation (SD) units, taking as reference the SD in the control group. Those variables with adjusted *p*-value < 0.05 and a minimum of 0.5 absolute size effect were selected. In the case of highly correlated variables (Pearson’s r > 0.8), only that with the maximum absolute size effect was kept. For chemokine and cytokine comparative analysis, the Mann–Whitney–Wilcoxon test was used to detect statistically significant differences (*p*-value < 0.05) and the binary logarithm of fold-change (the mean of the case group divided by the mean of the control group) to estimate the size effect.

### 2.8. COVID-19 Model

An Orthogonal partial least squares discriminant analysis (O-PLS-DA) was performed between HC cohort and AC (0 and 1) samples at t = 0 using selected variables as input, after being log-transformed and standardized (mean centering and unit variance). The O-PLS-DA implementation was in the metabom8 (v. 0.4.4, https://github.com/tkimhofer/metabom8, accessed on 15 July 2022) R package, combined with in-house R scripts for performance evaluation. A model of two components (one predictive t_pred_ and one orthonormal t_orth_) was trained through a five-fold cross-validation process repeated 10 times. A receiver operating characteristic (ROC) curve analysis based on t_pred_ component from training was used to determine the optimal cutoff according to Youden’s index. The area under the ROC curve (AUC), sensitivity and specificity values were also calculated from this analysis. The statistical significance of the different metrics (AUC, Sensitivity and Specificity) was estimated through permutation tests (100 runs).

### 2.9. Estimation of Recovery Days

It was performed for those individuals with acute COVID-19 and at least one follow-up sample with a minimum of 14 days since COVID-19. For each individual meeting this criterion, a linear model was built with t_pred_ as the dependent variable and the number of days since COVID-19 as the independent one. The slope and intercept of each model were used to calculate the required number of days to reach the average t_pred_ of the HC group. The obtained distribution of recovery days was modeled as a Generalized Extreme Value (GEV) distribution with evd (v. 2.3-6, https://cran.r-project.org/package=evd, accessed on 1 August 2022) R package to obtain their parameters of location, scale and shape.

## 3. Results

### 3.1. Cohorts under Consideration

**Figure 1:**
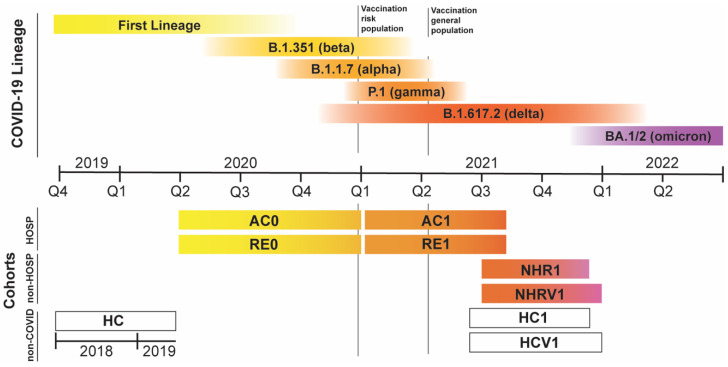
The COVID-19 disease time evolution, showing the approximate duration of the different lineages of the virus and the times when serum samples from the different cohorts were collected. The COVID-19 status and general characteristics of the cohorts are described in Table 1 and Tables S1–S3, respectively.

To investigate the natural history of COVID-19 disease, we have obtained serum samples from several hospitals in Spain (Basque Country and Madrid) to build up cohorts that represent many of the potential scenarios of the disease (Table 1 and Figure 1). In addition, we also collected samples from COVID-19-free subjects: samples collected before 2020 (HC cohort) [18] and samples collected in 2021 from unvaccinated and vaccinated subjects that reported not having passed the disease (HC1 and HCV1 cohorts, respectively, Table 1). To account for the severe infection cases, we have obtained serum samples from hospitalized patients as close as possible to the onset of the disease (D0-7): cohorts AC0 and AC1, collected during 2020, and 2021. To investigate the metabolic and immunologic normalization of severe patients, we expanded the AC0 and AC1 cohorts with longitudinal studies that covered the recovery phase for 490 of these individuals: up to three samples within sixty days from the onset of the disease and one sample beyond sixty days (RE0 and RE1 cohorts, Table 1). Finally, we also collected samples obtained in the medical check-up of the employees from a large corporation that include non-hospitalized patients who self-reported having passed COVID-19 in the past (at least 7 days after the onset of the disease, referred to herein as non-hospitalized recovered, NHR). Since the COVID-19 onset date and vaccination status are known for these donors, cohorts are divided into NHR1 and NHRV1 for the unvaccinated and vaccinated patients, respectively (Table 1 and Figure 1). Of note, of the vaccinated people (n = 2740) 15.3% got COVID-19 as compared to the 28.5% of the unvaccinated donors (n = 333). General characteristics and metadata for the different cohorts are presented in Tables S1–S3.

Serum samples were collected and analyzed using strict standard operating procedures to minimize sample biases (see Section 2). These procedures included the use of samples without heat-inactivation, restriction to only one thawing step and immediate sample measurement after thawing (<5 h) [19]. Serum analysis for the entire dataset included metabolite quantification and lipoprotein profile characterization by NMR spectroscopy; GlycA and GlycB quantitative determination [20] and the supramolecular phospholipid composite (SPC) cardiovascular risk associated marker quantification [21]. For a selected subset of the samples (226 samples in total), a representative panel of inflammation markers (cytokines and chemokines, see Section 2) [22,23,24,25] was also determined. A fraction of the samples for the prospective study of hospitalized patients have already been reported [2]. Samples belonging to different hospitals could not be discriminated in a PCA analysis (Figure S1), in line with the strict standard operating procedures used during sample recollection, preparation and measurement.

### 3.2. Early Metabolic Alterations in Acute and Mild COVID-19 Patients

**Figure 2:**
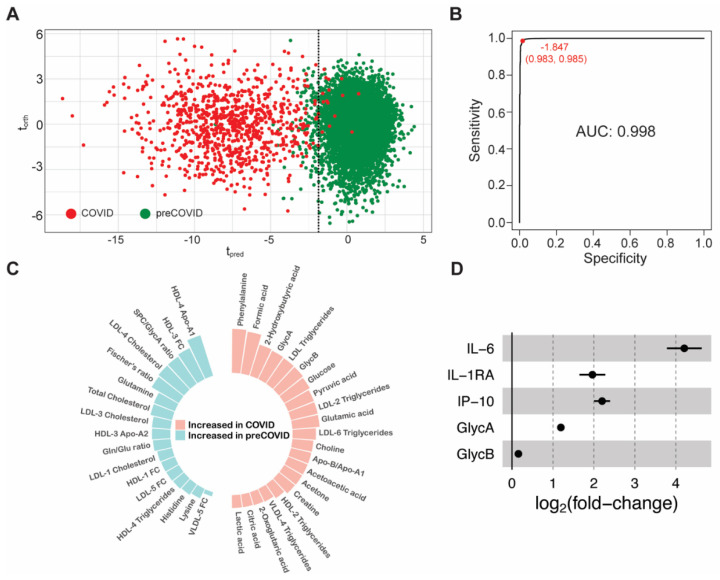
(**A**) Score plot from O-PLS-DA model that discriminates between COVID-19 patients (red circles, AC0 and AC1 cohorts) and healthy individuals (green circles, HC cohort). Dashed line corresponds to the value that maximizes Youden’s index. (**B**) Receiver operating characteristic curve for the O-PLS-DA model shown in Figure 2A. The area under the curve (AUC) is 0.998. In red it is indicated the t_pred_ value that maximizes the Youden’s index and the resulting specificity and sensitivity between parentheses. (**C**) Circular bar plot with the 38 metabolic parameters used for the discrimination model. The bars are proportional to the weight in the discrimination. (**D**) Forest plot with some inflammation markers under consideration. Filled circles represent statistically significant differences (*p*-value < 0.05). Horizontal lines are standard errors.

COVID-19 cases that require hospitalization present a severe phenotype compared to mild or asymptomatic patients. Metabolic analysis of sera from hospitalized patients in the acute phase of the disease revealed many dysregulated metabolic pathways and an altered lipoprotein composition [2,5,7]. Using all the available samples that are close to the disease onset (cohorts AC0 and AC1), we generated a metabolic model that discriminates between COVID-19 patients and healthy individuals (Figure 2A). Consistent with previous observations [4,21,26,27], the metabolic alterations as measured by NMR spectroscopy can clearly separate severe COVID-19 patients from non-infected subjects, with an AUROC value of 0.998 (Figure 2B, Table S4). Indeed, only a subset of 38 parameters (obtained by univariate analysis) is needed for full discrimination between the two classes, including metabolites found upregulated in COVID-19 (phenylalanine, glucose, glutamate, choline, …); downregulated metabolites (glutamine, histidine, lysine, …); upregulated inflammation markers, and parameters that account for the lipoprotein profile rearrangement (Figure 2C). This complex alteration of metabolism is consistent with an elevated atherogenic risk, transient diabetes and liver damage, as previously reported [2,5,28].

The severity of the disease has been associated with a systemic exacerbated immune response [10]. For a subset of the acute cohort (n = 50), we measured a panel of 13 chemokines and cytokines already associated with the COVID-19 immunological response (see Materials and Methods and Figure S2) and compared them to equivalent determinations on the control cohort (n = 37). We found up to seven cytokine/chemokines significantly increased in hospitalized COVID-19 patients; with IL-1RA, IP-10 and IL-6 being about two-fold or higher upregulated as compared to the normal values measured in the healthy individuals (Figure 2D). These patients also showed a decrease in lymphocyte and monocyte counts, and an increase in neutrophils (Tables S1 and S2). Consistently, the GlycA and GlycB inflammation markers are also elevated (Figure 2D). Altogether, this immunological phenotype is consistent with an elevated risk of thrombus formation (IL-6) [29] and with an attempt of the body to decrease the inflammatory response caused by the infection with SARS-CoV-2 (elevated IL-1RA levels) [30].

Next, we investigated the samples from non-hospitalized recovered donors of previous COVID-19 infection (from the cohorts NHR1 and NHRV1). This cohort was validated by antigen testing and includes asymptomatic subjects as well as patients who self-reported compatible mild symptomatology at the time of the infection. Still, this cohort would not represent mild patients since the collected sample happened in the recovery phase. We decided not to discriminate between vaccinated and non-vaccinated donors because the impact of vaccination on our metabolic model is largely negligible (vide infra), so we refer to NHR1 + NHRV1 as NHR. Metabolomic serum analysis of this cohort revealed little variations from the control cohort (HC or HC1), indicating that this group does not develop a specific metabotype associated with the former SARS-CoV-2 infection meaning that, if a specific metabotype was developed near the onset, now it is no longer present. Moreover, when projected in the O-PLS-DA representation of the discrimination model for the COVID-19 acute patients, the NHR group falls in the region populated by healthy individuals (Figure S3). Finally, the associated inflammation markers (GlycA and GlycB) and the cytokine and chemokine quantification (Figure S4) did not report abnormal values for this cohort, except for MCP-1, which is significantly elevated (*p* = 0.022).

### 3.3. Metabolic Phenoreversion over Time for Hospitalized and Non-Hospitalized Recovered Patients

**Figure 3:**
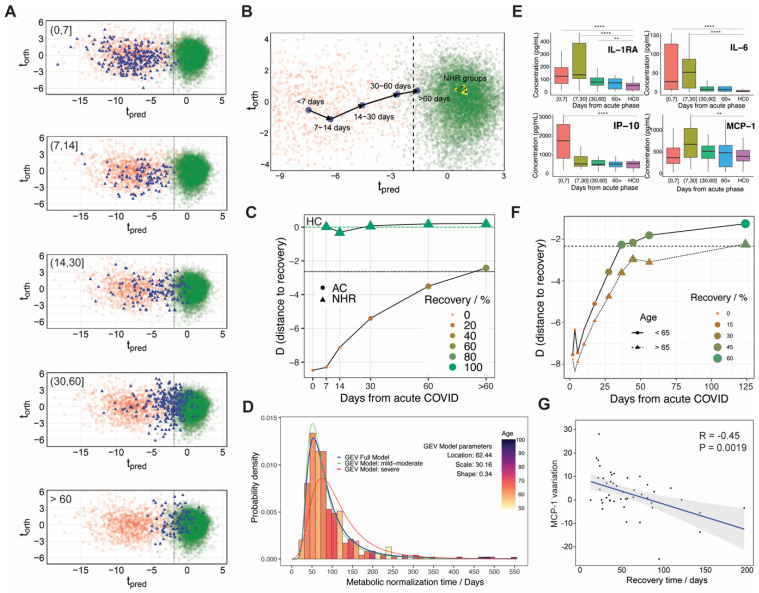
(**A**) Projection of the recovery samples (blue triangles, RE0 and RE1 cohorts) in the O-PLS-DA model that discriminates between COVID-19 patients (red circles, AC0 and AC1 cohorts) and healthy individuals (green circles, HC cohort), as a function of the recovery time for the sample, as indicated in the plots. (**B**) Projection of the recovery trajectory on the O-PLS-DA model of COVID-19 discrimination for hospitalized patients (blue circles) and non-hospitalized recovered (NHR) (yellow circles). (**C**) distance to recovery (D) as a function of the days from the infection onset for hospitalized patients (circles) and NHR individuals (triangles). The color and the size of the circles or triangles is proportional to the fraction of recovered people at a given time, as indicated in the legend. (**D**) Histogram of the individual recovery times for the metabolic phenotype assuming a linear recovery model. The distribution fits well to a GEV function (blue line), whose fitting parameters are enclosed. The green and red lines show the same type of model but using only a subset of patients according to the hospital’s severity criteria: mild-moderate (green) or severe (red). The color code for the histogram bars displays the average age, as indicated in the legend. (**E**) Boxplots showing the time evolution of serum concentration corresponding to the four cytokines/chemokines that were found significantly upregulated in hospitalized patients. Statistical significance of the difference between the two groups was estimated by the *p*-value: 0.01 (**) and 0.0001 (****). (**F**) distance to recovery (D) as a function of the days from the infection onset for hospitalized patients as a function of age: people above/below 65 years old are represented by circles/triangles. The color and the size of the circles or triangles are proportional to the fraction of recovered people at a given time, as indicated in the legend. (**G**) Correlation between the variation of MCP-1 over time (assuming a linear decay from day 8) and the individual recovery time (assuming linear recovery). The blue line corresponds to the best linear fit, with the confidence levels depicted in gray.

We have used the longitudinal recovery studies collected during 2020 and 2021 (combining cohorts RE0 and RE1) to investigate the metabolic restoration (or phenoreversion [11,12]) of COVID-19 hospitalized patients. Figure 3A,B show the projection on the O-PLS-DA model for COVID-19 discrimination of the recovery samples at different times. At the population level, this is a proper way to monitor COVID-19 metabolic phenoreversion: at short recovery times (t < 30 days) the metabotype ensemble overlaps with the acute phase one, while at longer times (t > 30 days) the metabotype ensemble migrates towards the distribution representing the healthy controls (HC). An important observation is that, on average, metabolic phenoreversion largely exceeds the hospitalization period taking place over several weeks, consistent with previous observations [11].

Metabolic normalization after COVID-19 infection of the ensemble can be also represented by using a quantitative parameter “D” that accounts for the statistical distance to the HC metabotype (D = t_pred_[t] − t_pred_[cutoff] + k, see Materials and Methods). Such distance is represented as a function of time in Figure 3C (circles), also showing the percentage of patients with normalized metabolism within the size and color of the circles. At short times, serum from hospitalized patients exhibits a large distance from the average HC metabotype, consistent with the associated metabolic dysregulation observed during acute infection. Yet, phenoreversion occurs for the hospitalized COVID-19 patient population at a close-to-exponential rate (on average), with about 50% recovery in 60 days. Such long times indicate that, on average, the hospitalized COVID-19 population is exposed to abnormal metabolite concentrations and a dysregulated lipoprotein profile for a time that may be long enough to significantly modify the atheroma plaque, providing an explanation for the recent observation that COVID-19 patients undergo an elevated risk of cardiovascular events [14]. At the metabolite level, phenoreversion occurs in a rather concerted way (Figure S5), with most of the altered metabolites and lipoproteins showing similar time-domain profiles (Figure S6).

It has been demonstrated that metabolic normalization is heterogeneous and it is largely patient-dependent [11]. In line with this observation, we have also analyzed the individual metabolic phenoreversion for the subjects that donated three or more samples (n = 351). Here, a linear model is used to calculate the metabolic recovery time for each subject which is represented in the histogram of Figure 3D. The comparison of the individual metabolic normalization times shows a skewed distribution, and the experimental recovery times can be adjusted to a generalized extreme value distribution (location, 62.44; scale, 30.16; shape, 0.34). The full model (blue line) also indicates a maximum probability for metabolic recovery in 62 days, with 95% of the population normalizing their metabolism before 284 days. Recovery is age-dependent and elder patients showed longer metabolic normalization times than younger ones (color code in Figure 3D). Consistently, the recovery function for the cohort is also age-dependent and subjects younger than 65 years old recover significantly faster than the elder ones (Figure 3F). Importantly, metabolic phenoreversion is also dependent on the severity of the disease and a clinical sub-classification of the acute patients (between severe and moderate/mild) also modulates the distributions, as shown in Figure 3D: green and red lines are sub-models that, respectively, take into account only patients that have been classified as mild-moderate or severe by hospitals (see Tables S1 and S2). The maximum probability of metabolic recovery for mild-moderate patients is 59 days, with 95% of the population with metabolic normalization before 258 days. For severe patients, the metabolic recovery period is 82 days, with 95% experiencing metabolic phenoreversion before 309 days.

We have also measured the time evolution of the panel of inflammation markers. As shown in Figure 3E and Figure S2, the levels of cytokines and chemokines that were upregulated progressively return to the values observed in the control cohort, within a similar timescale to the metabolic phenoreversion. In an equivalent mode as for metabolic phenoreversion, we have calculated the average half-recovery time for each inflammation marker, using a linear model. For MCP-1, we found a statistically significant correlation between the metabolic and inflammatory recovery times (Figure 3G), indicating that both events may be coupled temporally. Actually, MCP-1 is associated with triggering a metabolic response essential for the earliest cellular responses of atherogenesis [31].

Finally, the non-hospitalized recovered cohort did not report any metabolic fingerprint associated with infection at a timeframe within a month of the onset of the disease, indicating full metabolic normalization for this cohort during this time. In line with this observation, samples spanning at longer times from the disease onset always showed equivalent metabotypes to the HC cohort (Figure 3C, triangles).

### 3.4. On the Lineage-Specific Metabolic Response of SARS-CoV-2

**Figure 4:**
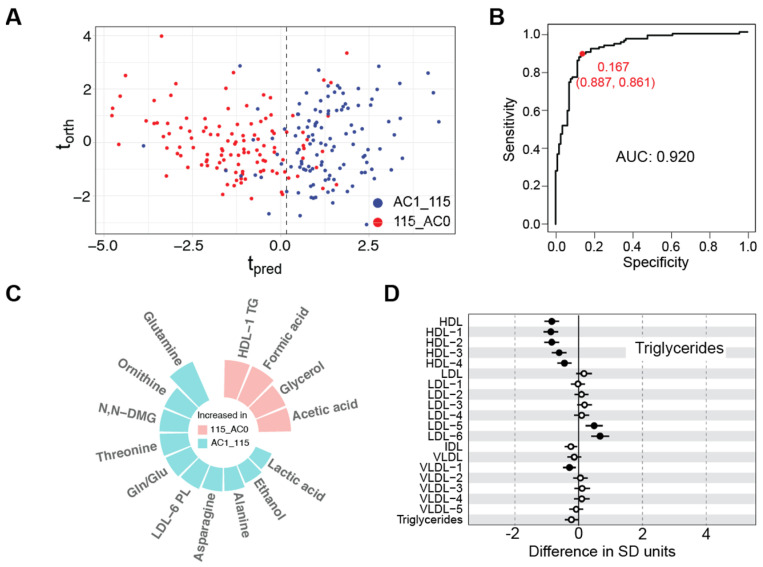
(**A**) O-PLS-DA score plot representation for the discrimination between the first 115 samples collected during 2020 (115_AC0, red circles) and the last 115 samples collected during 2021 (AC1_115, blue circles). (**B**) Receiver operating characteristic curve for the O-PLS-DA model shown in Figure 2A. The area under the curve (AUC) is 0.92 In red is indicated the t_pred_ value that maximizes Youden’s index and the resulting specificity and sensitivity between parentheses. (**C**) Circular bar plot with the 14 metabolic parameters used for the discrimination model. The bars are proportional to the weight in the discrimination. (**D**) Triglyceride variation between 115_AC0 and AC1_115.

Our longitudinal COVID-19 study spans more than one and a half years. During this time, the virus has mutated extensively with variants that significantly differ in virulence [32,33]. Figure 1 shows the SARS-CoV-2 lineage predominance in Spain according to the European Centre for Disease Prevention and Control (https://www.ecdc.europa.eu/en/covid-19/variants-concern, accessed on 1 September 2022). Even though it is very difficult to establish strict boundaries for the different lineages of the virus, it seems clear that the cohorts AC0 and RE0 mainly report on the effects of a subset of virus variants (first lineage, alpha and beta) while the cohorts AC1 and RE1 will be most influenced by the gamma and delta variants (Figure 1). To investigate the impact of the predominant virus lineage on the metabolism we have compared the first 115 samples collected from the AC0 cohort (115_AC0) with the last 115 ones collected in the AC1 cohort (AC1_115). Both datasets can be well discriminated and Figure 4A shows the O-PLS-DA representation of the discrimination model (see performance metrics in Table S5). Even though both datasets retain the characteristic fingerprint associated with COVID-19 infection when compared to HC, the AC1_115 and 115_AC0 cohorts can be discriminated due to specific changes in a set of metabolites and lipoproteins (Figure 4C), while no changes could be detected in the cytokine/chemokine profiles nor in the other inflammation markers (i.e., GlycA and GlycB). The characteristic triglyceride (TG) dysregulation (higher population of TG in the LDL subparticles for AC1_115, Figure 4D) could be associated with the highest virulence observed with the delta variant (B.1.617.2) as compared to the former lineages of the virus. In turn, the inflammation markers are mainly associated with the severity of the disease and all patients in the cohort were hospitalized under severe conditions.

### 3.5. Potential Confounding Factors and Limitations of the Study

Many factors other than SARS-CoV-2 infection may affect metabolism and they must be considered as putative confounding factors in the metabolomic analysis. Massive campaigns of vaccination were promoted as a response to the pandemic after 2021 (Figure 1). Most hospitalized patients (AC0 and AC1 cohorts) had not been vaccinated at the time when samples were obtained. Still, we can investigate the effect of vaccination in our metabolic model since we have access to the vaccination status for the general population cohorts (NHR1, NHRV1, HC1 and HCV1). On average, samples of the cohort HCV1 were obtained after 109 ± 55 days from the last vaccination of the donor. Under these conditions, a comparison between HC1 and HCV1 cohorts reflects the minimum perturbations in our metabolic model due to vaccination (Figure S7). Specifically, no significant variations in the lipoprotein profile can be attributed to vaccination and only a couple of metabolites (glutamine and glycerol) show a statistically significant variation. Figure S8 represents both metabolite levels as a function of the vaccine type to show that no differences are observed among the different vaccines and, in all cases, a very small decrease/increase in the glutamine/glycerol levels was observed when compared to non-vaccinated donors. It is important to emphasize that we do not claim that vaccination does not produce any alteration in the metabolism; our results just show that vaccination in the HCV1 cohort does not significantly alter our metabolic readout, most likely due to the large time that, on average, has passed after vaccination and sample collection.

Age and gender are also important factors that modulate metabolism and need to be considered. The selection of the 38 parameters within the univariate analysis considered this including age and gender as covariates. The final model, shown in Figure 2A, did not include age or gender as input parameters to avoid a potential bias. To demonstrate that metabolomics parameters can distinguish COVID-19 independently of age or gender, an alternative model was built using only matched sub-cohorts for the hospitalized COVID-19 and HC populations. This model renders equivalent specificity and sensitivity results (Figure S9, Table S6). Even if two separate models are built for each sex the resulting O-PLS-DA loadings of the predictive component remain with the same sign and very similar magnitude (Figure S10).

A large number of acute patients received medication right after admission to the hospital. Based on these premises, we compared the samples obtained at the hospitalization time (obtained before administration of any drug) with samples collected after two days of hospitalization (with patients already enrolled in medication treatments), changes in the metabolism or in the cytokine response were observed but with a much lower fold-change (Figures S11 and S12). Indeed, the changes observed fit well with the metabolic phenoreversion model (Figure 3). That said, we could not assess more specific tests to account for the effect of specific medications, and this must be considered a limitation of the study.

This study has other limitations. Additionally, even though most of the cohorts have a large number of samples, the time distribution of the samples is not optimal (Table 1), and so is the sampling for the inflammation markers. Finally, the control cohorts HC and HC1 do not correspond to the same patients that later on underwent the disease.

## 4. Discussion

Here, we have investigated the metabolic normalization of COVID-19 patients and, to a lesser extent, the attenuation of the inflammatory response as well, always considering different scenarios of the disease. Population analysis of acute patients that required hospitalization presented marked metabolic and lipoprotein dysregulation, as previously described [34]. Cytokine and chemokine analyses suggest that these alterations mirror the exacerbated immune response against SARS-CoV-2 infection, as expected [22]. The longitudinal studies (cohorts RE0 and RE1) allowed the investigation of the metabolic phenoreversion of the disease, with an average time of more than 62 days. Since COVID-19 dysregulates lipoproteins profiles in a way that increases the atherosclerotic risk (i.e., elevated ApoB/Apo100, reduced SPC signal, …) [21] such long recovery times raise the possibility that the serum-altered lipoprotein composition may inflict significant damage in the arteries, engrossing the atheroma plaque. A recent study demonstrates that post-COVID-19 patients have an elevated risk of undergoing cardiovascular episodes as well as other metabolic complications [35,36]. Our study provides a rationale for these observations.

At the individual level, it has been demonstrated that phenoreversion is largely heterogeneous in recovery time [11]. To account for such variability, we estimated the individual times for metabolic normalization using a linear model for phenoreversion. The distribution of such values indicates that age and disease severity are conditioning factors that ultimately affect metabolic phenoreversion: the recovery times for people above 65 years old or clinically classified as severe are significantly longer than average (Figure 3D,F). It is expected that other factors, such as some genetic variants, are associated with a severe response to SARS-COV-2 infection [8], and may also effectively modulate the recovery time for the metabolic response. Unfortunately, we do not have access to genetic data for our cohorts.

For those patients for whom we also had cytokine/chemokine analyses, we did an equivalent estimation of the decay in the inflammatory response. We could not find a correlation between the magnitude of the inflammatory response, with the exception of MCP-1 (Figure 3G), a chemokine that has been linked to the early response to atherogenesis [31]. Indeed, MCP-1 has been associated with oxidative stress and the level of circulating MCP-1 is significantly increased in type 1 and type 2 diabetes

Finally, the design of our longitudinal studies allowed separating patients according to the different variants of the virus, without complete discrimination, though. According to epidemiological data (Figure 1), AC0 and RE0 are dominated by the original strain and variants beta and alpha, while the AC1 and RE1 cohorts mainly provide information on people infected by the alpha, gamma and delta variants of the virus. The comparison between the two-time boundaries of the cohorts (115_AC0 versus AC1_115) reveals the impact of the virus evolution on the metabolic response: as expected, samples dominated by the gamma and delta variants produced a more severe metabolic dysregulation, affecting mostly the fold-change in lipoproteins and metabolites.

In summary, we have investigated the metabolic natural history of COVID-19 disease to find that the metabolic phenoreversion largely exceeds in time to the physical recovery (symptomatology). This situation provides an explanation for the elevated atherosclerotic risk observed in post-COVID-19 cohorts. Future work will focus on the long-term effects (2 or more years after COVID) by monitoring cases of long COVID-19 and correlations with associated cardiovascular events.

## Figures and Tables

**Table 1:** COVID-19 and vaccination status for the cohorts under consideration.

Donor Type	Cohort	Days from Disease Onset	N	Recollection Time	Expected COVID-19 Variants	Vaccinated
Hospitalized COVID-19 patients	AC0	0	697	Apr–Dec 2020	FL, α, β	No
	RE0	(0,7], (7–14], (14–30], (30,60], >60, TOT	137, 96, 104, 97, 10, 444		FL, α, β	No
	AC1	0	189	Jan–Oct 2021	γ, δ	Mixed
	RE1	(0,7], (7–14], (14–30], (30,60], >60, TOT	100,1,15, 158, 79, 353		γ, δ	Mixed
General population	HC	no COVID	8664	Before 2020	none	No
	NHR1	(7–14], (30,60], >60, TOT	1, 16, 78, 95	Jun–Nov 2021	δ, ο	No
	NHRV1	(0,7], (7–14], (14–30], (30,60], >60, TOT	1, 2, 8, 27, 380, 418	Jun–Dec 2021	δ, ο	Yes
	HC1	no COVID	238	May–Nov 2021	none	No
	HCV1	no COVID	2322	May–Dec 2021	none	Yes

Abbreviations: TOT, total number; FL, first Lineage; α, alpha/B1.1.7; β, beta/B.1.351, γ, gamma/P.1, δ, delta/B1.617.2; ο, omicron/BA.1/2.

## Data Availability

The data presented in this study will be available with publication from the corresponding authors upon reasonable request. Data is not publicly available due to privacy.

## Supplementary Materials

The following supporting information can be downloaded at: https://www.mdpi.com/article/10.3390/metabo12121206/s1, Table S1: General characteristics and metadata for the different cohorts of COVID-19 hospitalized patients whose disease onset was in 2020; Table S2: General characteristics and metadata for the different cohorts of COVID-19 hospitalized patients whose disease onset was in 2021; Table S3: General characteristics and metadata for the different cohorts of healthy controls (HC, HC1 and HCV1) and people that non-hospitalized recovered individuals (NHR1, NHRV1); Table S4: Main performance metrics for COVID-19 model; Table S5: Main performance metrics for COVID-19 lineage model; Table S6: Main performance metrics for age-gender balanced COVID-19 model; Figure S1: Principal Component Analysis (PCA) score plot showing the first and second components that explains the maximum variability (19.7% and 15.1%, respectively) of metabolomics data from acute patients at different hospitals; Figure S2: Concentration of chemokines and cytokines that are already associated to the COVID-19 immunological response in different time periods from disease onset (in days) and compared to healthy cohort (HC); Figure S3: O-PLS-DA score plot from COVID-19 model with the projection of non-hospitalized recovered (NHR) people (triangles); Figure S4: Comparison of inflammation markers, cytokine and chemokine levels of non-hospitalized recovered (NHR) group within 1.5 months from COVID-19 onset versus controls (HC); Figure S5: Concentration evolution for the 38 metabolomic parameters used to build the COVID-19 model. Evolution goes from acute phase (AC) through the different recovery periods (RE); Figure S6: Comparison of acute COVID-19 and the different recovery stages versus the control group (HC) for each analyzed metabolic parameter; Figure S7: Effect of vaccination between HCV1 and HC1 cohorts for each analyzed metabolic parameter; Figure S8: Violin plots showing the effect of vaccines by type for the concentration of Glutamine and Glycerol compared to people without vaccination (none); Figure S9: COVID-19 model using a subset of acute COVID-19 (AC) and control cohorts (HC) that have been matched between them by gender and age; Figure S10: Comparison of O-PLS-DA loadings from predictive components of two COVID-19 models by gender: one using only women and other using only men; Figure S11: Estimation of the effect of medication in COVID-19 acute patients after 1–2 days at hospital as compared versus initial collected sample at hospitalization; Figure S12: Estimation of the effect of medication on cytokine and chemokine levels for COVID-19 acute patients after 1–2 days at hospital as compared versus initial collected sample at hospitalization.
