# Supplementary material for: An NMR-Based Model to Investigate the Metabolic Phenoreversion of COVID-19 Patients throughout a Longitudinal Study
Source: Metabolites. 2022 Dec 1;12(12):1206. doi: 10.3390/metabo12121206 (PMC9788519; doi:10.3390/metabo12121206)

Table S1. General characteristics and metadata for the different cohorts of COVID hospitalized patients whose disease onset was in 2020. Recovery phase (RE0) is divided by days after the disease onset.

	**AC0 Acute phase N=697**	**RE0 (0,7] N=139**	**(7,14] N=96**	**(14,30] N=104**	**(30,60] N=134**	**>60 N=37**	**N**
**General**							
Age (years)	67.5 (16.4)	68.3 (13.6)	69.0 (16.4)	67.1 (13.4)	65.8 (12.5)	68.3 (13.7)	1207
Gender (female, n)	319 (45.8%)	51 (36.7%)	38 (39.6%)	34 (32.7%)	45 (33.6%)	14 (37.8%)	1207
**COVID**							
COVID vaccine dosis							1207
0	697 (100%)	139 (100%)	96 (100%)	104 (100%)	134 (100%)	36 (97.3%)	
1	0 (0.00%)	0 (0.00%)	0 (0.00%)	0 (0.00%)	0 (0.00%)	0 (0.00%)	
2	0 (0.00%)	0 (0.00%)	0 (0.00%)	0 (0.00%)	0 (0.00%)	1 (2.70%)	
Hospital severity criteria							822
mild-moderate	262 (84.0%)	116 (83.5%)	86 (89.6%)	81 (77.9%)	103 (76.9%)	21 (56.8%)	
severe	50 (16.0%)	23 (16.5%)	10 (10.4%)	23 (22.1%)	31 (23.1%)	16 (43.2%)	
**Medical history**							
Cancer	17 (12.8%)	4 (7.14%)	10 (11.2%)	8 (10.7%)	2 (11.1%)	0 (.%)	371
Cardiovascular disease	157 (22.8%)	24 (17.3%)	18 (18.8%)	22 (21.2%)	20 (14.9%)	4 (10.8%)	1200
Cerebrovascular disease	58 (8.41%)	8 (5.76%)	2 (2.08%)	2 (1.92%)	11 (8.21%)	4 (10.8%)	1200
Chronic kidney disease	20 (15.0%)	7 (12.5%)	8 (8.99%)	10 (13.3%)	0 (0.00%)	0 (.%)	371
Chronic obstructive pulmonary disease	51 (7.39%)	11 (7.91%)	6 (6.25%)	4 (3.85%)	10 (7.46%)	1 (2.70%)	1200
Diabetes	129 (18.7%)	21 (15.1%)	16 (16.7%)	27 (26.0%)	13 (9.70%)	6 (16.2%)	1200
Digestive disease	15 (11.3%)	5 (8.93%)	12 (13.5%)	9 (12.0%)	0 (0.00%)	0 (.%)	371
Hepatitis B hevirus	1 (0.27%)	0 (.%)	0 (.%)	0 (.%)	0 (.%)	0 (.%)	377
Hypertension	317 (45.9%)	69 (49.6%)	49 (51.0%)	58 (55.8%)	52 (38.8%)	17 (45.9%)	1200
Immunodeficiency	18 (3.23%)	1 (1.20%)	1 (14.3%)	1 (3.45%)	1 (0.86%)	2 (5.41%)	829
Immunosuppression	7 (5.26%)	4 (7.14%)	6 (6.74%)	0 (0.00%)	0 (0.00%)	0 (.%)	371
Kidney failure	12 (3.83%)	6 (4.32%)	7 (7.29%)	8 (7.69%)	4 (2.99%)	2 (5.41%)	823
Liver failure	9 (1.62%)	1 (1.20%)	0 (0.00%)	1 (3.45%)	1 (0.86%)	0 (0.00%)	829
Lymphadenopathy	0 (0.00%)	0 (0.00%)	0 (0.00%)	0 (0.00%)	0 (0.00%)	0 (0.00%)	452
Neoplasm	39 (9.07%)	5 (6.02%)	1 (14.3%)	1 (3.45%)	6 (5.17%)	1 (2.70%)	702
Obesity	16 (12.1%)	6 (10.7%)	10 (11.2%)	14 (18.7%)	5 (27.8%)	0 (.%)	370
Renal insufficiency	28 (7.43%)	0 (.%)	0 (.%)	0 (.%)	0 (.%)	0 (.%)	377
**Biochemical**							
Albumin (g/dL)	3.72 (0.46)	3.40 (0.51)	2.90 (.)	3.97 (0.35)	4.25 (0.88)	3.78 (1.73)	366
Alanine transaminase (U/L)	39.4 (41.6)	56.3 (42.5)	75.7 (31.0)	28.7 (7.81)	24.4 (10.8)	38.6 (57.2)	605
Activated partial thromboplastin time (s)	24.4 (4.94)	-	-	-	-	-	245
Aspartate transaminase (U/L)	59.6 (39.5)	61.0 (32.0)	37.5 (19.1)	22.6 (9.81)	21.1 (8.36)	23.0 (6.97)	285
Bilirubin (mg/dL)	0.55 (0.36)	0.43 (0.23)	0.37 (0.06)	0.40 (0.12)	0.71 (0.44)	0.61 (0.32)	467
C-reactive protein (mg/L)	74.4 (67.7)	39.4 (37.7)	17.8 (16.1)	3.83 (5.09)	6.72 (17.2)	3.75 (7.44)	758
Creatine kinase (U/L)	115 (156)	98.7 (201)	30.0 (12.2)	-	67.9 (42.5)	72.1 (54.8)	271
Creatine phosphokinase (U/L)	134 (227)	-	-	-	-	-	288
Creatinine (mg/dL)	0.96 (0.48)	0.85 (0.32)	0.66 (0.20)	1.07 (0.37)	0.82 (0.19)	0.84 (0.25)	613
D-dimer (ng/mL)	1824 (6768)	1067 (1680)	743 (229)	757 (489)	748 (1356)	915 (983)	722
Ferritin (ng/mL)	775 (799)	1293 (1113)	886 (475)	408 (274)	279 (240)	202 (201)	707
Glucose (mg/dL)	139 (93.2)	133 (58.1)	122 (51.8)	119 (35.0)	113 (36.5)	109 (40.3)	549
Interleukin-6 (pg/mL)	18.8 (23.9)	748 (1005)	-	-	-	-	15
Lactate dehydrogenase (U/L)	303 (164)	314 (94.0)	285 (148)	207 (31.1)	207 (44.4)	208 (56.5)	729
Leukocytes (10^9/L)	99.5 (925)	7.53 (3.31)	8.57 (2.40)	6.02 (1.73)	7.35 (3.83)	6.81 (1.59)	760
Lymphocytes (10^9/L)	14.7 (142)	1.16 (0.66)	1.14 (0.43)	2.21 (0.83)	2.32 (1.01)	2.25 (0.72)	759
Monocytes (10^9/L)	0.43 (0.24)	0.56 (0.59)	0.65 (0.59)	0.55 (0.15)	0.78 (0.98)	0.61 (0.15)	691
Neutrophils (10^9/L)	79.9 (774)	5.79 (3.05)	6.72 (1.98)	2.99 (1.57)	4.07 (2.41)	3.70 (1.18)	757
Platelets (10^9/L)	221 (100)	259 (98.3)	328 (97.1)	211 (57.6)	254 (78.3)	224 (66.2)	754

	AC0 Acute phase	RE0 (0,7]	(7,14]	(14,30]	(30,60]	>60	N
Procalcitonin (ng/mL)	1.20 (11.5)	0.66 (3.85)	0.07 (0.01)	0.05 (0.02)	0.06 (0.04)	0.05 (0.03)	569
Protein (g/dL)	6.31 (0.72)	6.50 (0.14)	-	6.85 (0.47)	6.64 (0.40)	6.22 (0.93)	166
Prothrombin activity (%)	88.5 (19.9)	87.7 (15.3)	85.0 (30.0)	26.0 (.)	97.2 (4.35)	93.6 (16.5)	558
Urea (mg/dL)	44.2 (25.4)	50.4 (24.6)	45.3 (15.5)	42.2 (25.2)	34.6 (15.5)	41.3 (16.7)	553
**Hospitalization events and treatments**							
Acute respiratory distress syndrome	12 (9.02%)	8 (14.3%)	8 (8.99%)	17 (22.7%)	6 (33.3%)	0 (.%)	371
Bilateral alveolar infiltrates	103 (77.4%)	46 (82.1%)	68 (76.4%)	64 (85.3%)	18 (100%)	0 (.%)	371
Continuos Positive Airway Pressure	13 (2.52%)	0 (0.00%)	0 (0.00%)	0 (.%)	0 (.%)	0 (.%)	604
CURB-65	0.92 (0.95)	-	-	-	-	-	106
Extracorporeal membrane oxygenation	1 (0.47%)	0 (.%)	0 (.%)	0 (.%)	0 (.%)	0 (.%)	213
Hemofiltration	2 (0.95%)	0 (.%)	0 (.%)	0 (.%)	0 (.%)	0 (.%)	211
High-flow oxygen therapy	36 (20.0%)	17 (20.5%)	3 (42.9%)	3 (10.3%)	21 (18.1%)	16 (43.2%)	452
High flow nasal cannula	53 (10.3%)	11 (13.3%)	1 (20.0%)	0 (.%)	0 (.%)	0 (.%)	604
Intensive care unit required	47 (22.0%)	5 (6.02%)	2 (28.6%)	2 (6.90%)	3 (2.59%)	9 (24.3%)	486
Interstitial infiltrates	1 (0.75%)	1 (1.79%)	2 (2.25%)	2 (2.67%)	0 (0.00%)	0 (.%)	371
Intubation days	23.1 (17.9)	-	-	-	-	-	24
Dead	38 (9.90%)	0 (.%)	0 (.%)	0 (.%)	0 (.%)	0 (.%)	384
Mechanic ventilation	3 (2.26%)	1 (1.79%)	4 (4.49%)	9 (12.0%)	9 (50.0%)	0 (.%)	371
Multi organic failure	0 (0.00%)	0 (0.00%)	0 (0.00%)	0 (0.00%)	0 (0.00%)	0 (.%)	371
Nasal cannulas	271 (51.1%)	12 (14.5%)	2 (40.0%)	0 (.%)	0 (.%)	0 (.%)	618
Number of hospital days	9.52 (13.8)	9.87 (13.4)	12.3 (5.47)	6.24 (3.19)	7.95 (10.7)	12.8 (11.2)	836
Orotracheal intubation	5 (2.81%)	1 (1.20%)	0 (0.00%)	0 (.%)	0 (.%)	0 (.%)	266
Oxygen therapy	120 (67.4%)	20 (24.1%)	2 (40.0%)	0 (.%)	0 (.%)	0 (.%)	266
Pneumonia:							693
bilateral	328 (77.9%)	76 (91.6%)	7 (100%)	26 (89.7%)	103 (88.8%)	33 (89.2%)	
normal	45 (10.7%)	4 (4.82%)	0 (0.00%)	0 (0.00%)	2 (1.72%)	1 (2.70%)	
unilateral	48 (11.4%)	3 (3.61%)	0 (0.00%)	3 (10.3%)	11 (9.48%)	3 (8.11%)	
SOFA score	2.37 (3.71)	-	-	-	-	-	19
Unilateral alveolar infiltrates	12 (9.02%)	3 (5.36%)	7 (7.87%)	5 (6.67%)	0 (0.00%)	0 (.%)	371
Venturi mask	83 (15.8%)	3 (3.61%)	1 (20.0%)	0 (.%)	0 (.%)	0 (.%)	614
**Measures at hospitalization**							
Breathing frequency (n/min)	21.7 (8.02)	15.3 (2.31)	-	-	13.3 (1.15)	18.0 (.)	72
Diastolic blood pressure (mmHg)	77.3 (13.4)	75.0 (10.3)	68.6 (10.4)	75.9 (11.1)	73.6 (16.5)	78.6 (13.9)	678
Heart rate (n/min)	89.7 (17.7)	86.5 (17.0)	75.7 (10.5)	90.6 (17.6)	87.9 (17.0)	86.2 (17.1)	690
Systolic blood pressure (mmHg)	132 (21.7)	128 (18.0)	126 (21.0)	122 (14.6)	127 (18.8)	133 (20.5)	688
Temperature (ºC)	36.6 (0.87)	36.8 (0.77)	37.1 (0.76)	37.0 (0.66)	36.9 (0.84)	36.6 (0.59)	686
**Symptoms at hospitalization**							
Ageusia	1 (0.75%)	1 (1.79%)	0 (0.00%)	0 (0.00%)	0 (0.00%)	0 (.%)	371
Anosmia	1 (0.75%)	1 (1.79%)	0 (0.00%)	0 (0.00%)	0 (0.00%)	0 (.%)	371
Arthralgia	0 (0.00%)	0 (0.00%)	0 (0.00%)	0 (0.00%)	0 (0.00%)	0 (.%)	371
Asthenia	7 (5.26%)	2 (3.57%)	3 (3.37%)	0 (0.00%)	0 (0.00%)	0 (.%)	371
Clouding of consciousness	19 (7.60%)	0 (.%)	0 (.%)	0 (.%)	0 (.%)	0 (.%)	250
Conjunctival congestion	2 (0.47%)	0 (0.00%)	0 (0.00%)	0 (0.00%)	0 (0.00%)	0 (0.00%)	702
Cough	112 (62.2%)	54 (65.1%)	1 (14.3%)	18 (62.1%)	76 (65.5%)	23 (62.2%)	452
Diarrhea	80 (20.9%)	2 (3.57%)	6 (6.74%)	5 (6.67%)	1 (5.56%)	0 (.%)	621
Disorientation	30 (5.46%)	4 (4.82%)	0 (0.00%)	0 (0.00%)	3 (2.59%)	2 (5.41%)	821
Dry cough	146 (38.1%)	4 (7.14%)	8 (8.99%)	0 (0.00%)	0 (0.00%)	0 (.%)	621
Dyspnoea	15 (11.3%)	5 (8.93%)	11 (12.4%)	3 (4.00%)	0 (0.00%)	0 (.%)	371
Expectoration	7 (5.26%)	0 (0.00%)	7 (7.87%)	2 (2.67%)	0 (0.00%)	0 (.%)	371
Fatigue	190 (44.2%)	15 (18.1%)	2 (28.6%)	8 (27.6%)	28 (24.1%)	10 (27.0%)	702
Fever	341 (60.6%)	78 (56.1%)	21 (21.9%)	31 (29.8%)	97 (72.4%)	29 (78.4%)	1073
Headache	72 (12.8%)	8 (5.76%)	1 (1.04%)	3 (2.88%)	16 (11.9%)	2 (5.41%)	1073
Hemoptysis	1 (0.23%)	0 (0.00%)	0 (0.00%)	0 (0.00%)	0 (0.00%)	0 (0.00%)	702
Loss of consciousness	4 (2.22%)	3 (3.61%)	0 (0.00%)	0 (0.00%)	3 (2.59%)	1 (2.70%)	452

	AC0	RE0					
	Acute phase	(0,7]	(7,14]	(14,30]	(30,60]	>60	N
Lymphadenopathy	2 (0.80%)	0 (.%)	0 (.%)	0 (.%)	0 (.%)	0 (.%)	250
Myalgia	119 (21.1%)	16 (11.5%)	7 (7.29%)	12 (11.5%)	24 (17.9%)	7 (18.9%)	1073
Nasal congestion	17 (3.02%)	3 (2.16%)	0 (0.00%)	1 (0.96%)	2 (1.49%)	3 (8.11%)	1073
Nausea	12 (6.67%)	6 (7.23%)	0 (0.00%)	2 (6.90%)	7 (6.03%)	4 (10.8%)	452
Nausea vomiting	40 (10.5%)	0 (0.00%)	2 (2.25%)	0 (0.00%)	0 (0.00%)	0 (.%)	620
Odynophagia	44 (10.2%)	8 (9.64%)	0 (0.00%)	4 (13.8%)	9 (7.76%)	0 (0.00%)	702
Oropharyngeal congestion	6 (1.40%)	3 (3.61%)	0 (0.00%)	0 (0.00%)	4 (3.45%)	0 (0.00%)	702
Productive cough	55 (22.0%)	0 (.%)	0 (.%)	0 (.%)	0 (.%)	0 (.%)	250
Shaking chills	75 (13.3%)	19 (13.7%)	0 (0.00%)	4 (3.85%)	9 (6.72%)	4 (10.8%)	1073
Skin rash	3 (1.20%)	0 (.%)	0 (.%)	0 (.%)	0 (.%)	0 (.%)	250
Sore throat	1 (0.75%)	0 (0.00%)	0 (0.00%)	0 (0.00%)	0 (0.00%)	0 (.%)	371
Tonsil inflammation	0 (0.00%)	0 (.%)	0 (.%)	0 (.%)	0 (.%)	0 (.%)	250
Vomiting	15 (8.33%)	6 (7.23%)	0 (0.00%)	1 (3.45%)	10 (8.62%)	4 (10.8%)	452

Table S2. General characteristics and metadata for the different cohorts of COVID hospitalized patients whose disease onset was in 2021. Recovery phase (RE1) is divided by days after the disease onset.

	AC1	RE1					
	Acute phase	(0,7]	(7,14]	(14,30]	(30,60]	>60	N
	N=189	N=100	N=1	N=15	N=121	N=52	
**General**							
Age (years)	61.4 (13.7)	62.9 (13.7)	70.0 (.)	61.9 (12.9)	62.9 (12.9)	57.4 (15.0)	478
Gender (female, n)	69 (36.5%)	36 (36.0%)	0 (0.00%)	5 (33.3%)	44 (36.4%)	21 (40.4%)	478
**COVID**							
COVID vaccine dosis							478
0	183 (96.8%)	98 (98.0%)	1 (100%)	15 (100%)	108 (89.3%)	48 (92.3%)	
1	6 (3.17%)	2 (2.00%)	0 (0.00%)	0 (0.00%)	7 (5.79%)	4 (7.69%)	
2	0 (0.00%)	0 (0.00%)	0 (0.00%)	0 (0.00%)	6 (4.96%)	0 (0.00%)	
Hospital severity criteria							478
mild-moderate	146 (77.2%)	77 (77.0%)	1 (100%)	11 (73.3%)	109 (90.1%)	26 (50.0%)	
severe	43 (22.8%)	23 (23.0%)	0 (0.00%)	4 (26.7%)	12 (9.92%)	26 (50.0%)	
**Medical history**							
Cardiovascular disease	22 (11.6%)	13 (13.0%)	0 (0.00%)	1 (6.67%)	18 (14.9%)	3 (5.77%)	478
Cerebrovascular disease	10 (5.29%)	7 (7.00%)	0 (0.00%)	0 (0.00%)	8 (6.61%)	2 (3.85%)	478
Chronic obstructive pulmonary disease	6 (3.17%)	6 (6.00%)	0 (0.00%)	1 (6.67%)	4 (3.31%)	1 (1.92%)	478
Diabetes	31 (16.4%)	17 (17.0%)	0 (0.00%)	5 (33.3%)	19 (15.7%)	5 (9.62%)	478
Hypertension	66 (34.9%)	36 (36.0%)	1 (100%)	3 (20.0%)	47 (38.8%)	15 (28.8%)	478
Immunodeficiency	3 (1.59%)	2 (2.00%)	0 (0.00%)	1 (6.67%)	1 (0.83%)	1 (1.92%)	478
Kidney failure	4 (2.12%)	4 (4.00%)	0 (0.00%)	1 (6.67%)	2 (1.65%)	1 (1.92%)	478
Liver failure	4 (2.12%)	1 (1.00%)	0 (0.00%)	1 (6.67%)	2 (1.65%)	1 (1.92%)	478
Lymphadenopathy	0 (0.00%)	0 (0.00%)	0 (0.00%)	0 (0.00%)	0 (0.00%)	0 (0.00%)	478
Neoplasm	12 (6.35%)	8 (8.00%)	1 (100%)	0 (0.00%)	7 (5.79%)	4 (7.69%)	478
**Biochemical**							
Albumin (g/dL)	3.83 (0.31)	3.52 (0.62)	-	3.96 (0.26)	4.55 (0.94)	4.56 (0.34)	353
Alanine transaminase (U/L)	47.5 (68.6)	69.1 (148)	-	35.0 (11.5)	24.8 (19.4)	26.0 (18.3)	399
Aspartate transaminase (U/L)	61.3 (89.2)	81.9 (143)	21.0 ()	20.4 (5.58)	21.9 (9.66)	22.7 (9.85)	160
Bilirubin (mg/dL)	0.53 (0.51)	0.94 (1.46)	-	0.80 (0.44)	0.65 (0.23)	0.50 (0.41)	167
C-reactive protein (mg/L)	60.4 (50.1)	29.3 (29.7)	2.50 (-)	4.46 (2.94)	7.06 (17.4)	3.97 (4.71)	461
Creatine kinase (U/L)	96.0 (92.9)	47.4 (38.0)	-	47.7 (24.5)	75.3 (44.8)	95.4 (47.7)	362
Creatinine (mg/dL)	0.85 (0.75)	0.75 (0.19)	-	0.99 (0.22)	1.87 (9.14)	0.84 (0.21)	403
D-dimer (ng/mL)	1120 (4516)	979 (1166)	950 (.)	407 (192)	588 (490)	467 (453)	254
Ferritin (ng/mL)	964 (1007)	1496 (1426)	857 (.)	412 (427)	253 (183)	203 (242)	234
Glucose (mg/dL)	143 (63.2)	125 (55.1)	-	127 (55.7)	114 (30.7)	113 (48.6)	396
Interleukin-6 (pg/mL)	37.5 (55.4)	34.7 (66.2)	-	5.00 (.)	-	6.00 (.)	25
Lactate dehydrogenase (U/L)	299 (86.4)	285 (99.9)	234 (.)	226 (55.8)	201 (46.5)	195 (31.1)	444
Leukocytes (10^9/L)	7.51 (7.23)	8.72 (7.34)	6.66 (.)	6.66 (1.91)	7.23 (6.96)	6.56 (1.63)	462
Lymphocytes (10^9/L)	1.46 (5.65)	1.95 (5.84)	3.74 (.)	2.07 (0.54)	4.29 (19.5)	2.18 (0.77)	462
Monocytes (10^9/L)	0.50 (0.75)	0.57 (0.27)	0.70 (.)	0.69 (0.32)	0.63 (0.26)	0.55 (0.14)	462

	AC1	RE1					
	Acute phase	(0,7]	(7,14]	(14,30]	(30,60]	>60	N
Neutrophils (10^9/L)	7.21 (18.4)	6.16 (2.97)	2.04 (.)	3.72 (1.62)	3.80 (1.81)	3.62 (1.19)	462
Platelets (10^9/L)	225 (96.3)	262 (108)	316 (.)	166 (68.5)	250 (71.5)	244 (75.2)	462
Procalcitonin (ng/mL)	0.16 (0.43)	0.07 (0.07)	0.06 (.)	0.05 (0.02)	0.04 (0.03)	0.04 (0.03)	434
Protein (g/dL)	5.90 (1.26)	5.65 (0.35)	-	6.60 (0.85)	6.88 (0.49)	6.65 (0.49)	27
Prothrombin activity (%)	91.5 (17.8)	93.6 (15.3)	-	96.0 (6.93)	91.6 (21.2)	95.9 (7.10)	371
Urea (mg/dL)	40.8 (17.0)	46.6 (18.5)	-	42.8 (24.5)	32.9 (10.4)	34.8 (11.8)	401
**Hospitalization events and treatments**							
Acute respiratory distress syndrome	2 (1.06%)	0 (0.00%)	0 (.%)	0 (0.00%)	0 (.%)	0 (.%)	287
High-flow oxygen therapy	43 (22.8%)	23 (23.0%)	0 (0.00%)	4 (26.7%)	12 (9.92%)	26 (50.0%)	478
High flow nasal cannula	42 (22.2%)	19 (19.6%)	0 (.%)	1 (100%)	0 (.%)	0 (.%)	287
Intensive care unit required	28 (14.8%)	17 (17.0%)	0 (0.00%)	3 (20.0%)	6 (4.96%)	18 (34.6%)	478
Nasal cannulas	149 (78.8%)	40 (41.2%)	0 (.%)	1 (100%)	0 (.%)	0 (.%)	287
Number of hospital days	8.54 (9.17)	9.73 (11.2)	4.00 (.)	10.0 (7.92)	6.82 (8.20)	11.6 (9.20)	478
Orotracheal intubation	5 (2.65%)	1 (1.03%)	0 (.%)	1 (100%)	0 (.%)	0 (.%)	287
Oxygen therapy	151 (79.9%)	42 (43.3%)	0 (.%)	1 (100%)	0 (.%)	0 (.%)	287
Pneumonia:							478
bilateral	161 (85.2%)	78 (78.0%)	1 (100%)	14 (93.3%)	97 (80.2%)	49 (94.2%)	
normal	5 (2.65%)	3 (3.00%)	0 (0.00%)	1 (6.67%)	3 (2.48%)	0 (0.00%)	
unilateral	23 (12.2%)	19 (19.0%)	0 (0.00%)	0 (0.00%)	21 (17.4%)	3 (5.77%)	
Venturi mask	35 (18.5%)	12 (12.4%)	0 (.%)	1 (100%)	0 (.%)	0 (.%)	287
**Measures at hospitalization**							
Breathing frequency (n/min)	23.9 (6.31)	25.0 (5.66)	-	-	27.2 (7.05)	23.0 (4.24)	24
Diastolic blood pressure (mmHg)	72.3 (21.5)	72.0 (23.3)	73.0 (.)	68.6 (31.7)	73.8 (20.8)	70.7 (20.5)	452
Heart rate (n/min)	95.2 (98.6)	89.4 (17.6)	110 (.)	84.5 (19.2)	86.6 (14.8)	119 (186)	462
Systolic blood pressure (mmHg)	133 (48.4)	138 (63.9)	117 (.)	136 (19.1)	130 (21.8)	139 (85.4)	446
Temperature (ºC)	36.8 (0.80)	36.9 (0.75)	37.2 (.)	36.9 (0.87)	36.7 (0.71)	36.9 (0.95)	447
**Symptoms**							
Conjunctival congestion	0 (0.00%)	0 (0.00%)	0 (0.00%)	0 (0.00%)	0 (0.00%)	0 (0.00%)	478
Cough	135 (71.4%)	71 (71.0%)	1 (100%)	8 (53.3%)	93 (76.9%)	31 (59.6%)	478
Disorientation	4 (2.12%)	1 (1.00%)	0 (0.00%)	0 (0.00%)	4 (3.31%)	1 (1.92%)	478
Fatigue	55 (29.1%)	34 (34.0%)	0 (0.00%)	4 (26.7%)	37 (30.6%)	15 (28.8%)	478
Fever	150 (79.4%)	84 (84.0%)	1 (100%)	10 (66.7%)	97 (80.2%)	41 (78.8%)	478
Headache	27 (14.3%)	15 (15.0%)	0 (0.00%)	0 (0.00%)	18 (14.9%)	10 (19.2%)	478
Hemoptysis	1 (0.53%)	1 (1.00%)	0 (0.00%)	0 (0.00%)	0 (0.00%)	1 (1.92%)	478
Loss of consciousness	4 (2.12%)	0 (0.00%)	0 (0.00%)	0 (0.00%)	2 (1.65%)	2 (3.85%)	478
Myalgia	40 (21.2%)	27 (27.0%)	0 (0.00%)	3 (20.0%)	25 (20.7%)	11 (21.2%)	478
Nasal congestion	2 (1.06%)	2 (2.00%)	0 (0.00%)	0 (0.00%)	1 (0.83%)	1 (1.92%)	478
Nausea	15 (7.94%)	5 (5.00%)	1 (100%)	2 (13.3%)	9 (7.44%)	4 (7.69%)	478
Odynophagia	13 (6.88%)	7 (7.00%)	1 (100%)	2 (13.3%)	6 (4.96%)	3 (5.77%)	478
Oropharyngeal congestion	2 (1.06%)	1 (1.00%)	0 (0.00%)	0 (0.00%)	0 (0.00%)	1 (1.92%)	478
Shaking chills	19 (10.1%)	11 (11.0%)	0 (0.00%)	1 (6.67%)	13 (10.7%)	4 (7.69%)	478

	AC1	RE1					
	Acute phase	(0,7]	(7,14]	(14,30]	(30,60]	>60	N
Vomiting	7 (3.70%)	2 (2.00%)	0 (0.00%)	0 (0.00%)	6 (4.96%)	1 (1.92%)	478

Table S3. General characteristics and metadata for the different cohorts of healthy controls (HC, HC1 and HCV1) and people that non-hospitalized recovered individuals (NHR1, NHRV1).

	**HC** N=8664	**HC1** N=238	**HCV1** N=2322	**NHR1** N=95	**NHRV1** N=418	**N**
**General**						
Age (years)	43.3 (9.18)	36.6 (9.26)	44.7 (9.17)	37.5 (10.3)	42.9 (10.3)	11737
Gender (female, n)	3172 (36.6%)	131 (55.0%)	963 (41.5%)	39 (41.1%)	182 (43.5%)	11737
Smoker	1744 (20.2%)	62 (26.1%)	470 (20.3%)	17 (18.3%)	48 (11.6%)	11683
**Measures**						
Body mass index (kg/m²)	25.4 (3.81)	24.2 (3.95)	25.4 (4.58)	24.8 (3.95)	25.6 (4.17)	11567
Height (cm)	172 (9.01)	170 (9.42)	171 (9.10)	171 (9.25)	171 (9.28)	11593
Weight (kg)	75.5 (14.2)	70.3 (14.0)	74.6 (15.1)	73.0 (14.0)	75.4 (14.9)	11600
**Questionnaire**						
Diabetes	73 (3.13%)	0 (.%)	0 (.%)	0 (.%)	0 (.%)	2335
Hypertension	955 (11.6%)	3 (1.29%)	155 (6.75%)	5 (5.32%)	33 (7.97%)	11279
Do physical exercise	6485 (75.2%)	188 (79.0%)	1764 (76.6%)	77 (84.6%)	329 (79.5%)	11664
**COVID**						
Days from COVID onset						513
(0,7]	-	-	-	0 (0.00%)	1 (0.24%)	
(7,14]	-	-	-	1 (1.05%)	2 (0.48%)	
(14,30]	-	-	-	0 (0.00%)	8 (1.91%)	
(30,60]	-	-	-	16 (16.8%)	27 (6.46%)	
>60	-	-	-	78 (82.1%)	380 (90.9%)	
COVID vaccine dosis						11489
0	8416 (100%)	238 (100%)	0 (0.00%)	95 (100%)	0 (0.00%)	
1	0 (0.00%)	0 (0.00%)	471 (20.3%)	0 (0.00%)	340 (81.3%)	
2	0 (0.00%)	0 (0.00%)	1851 (79.7%)	0 (0.00%)	78 (18.7%)	
Vaccine: Astra Zeneca	0 (0.00%)	0 (0.00%)	101 (4.35%)	0 (0.00%)	10 (2.39%)	11737
Vaccine: Janssen	0 (0.00%)	0 (0.00%)	209 (9.00%)	0 (0.00%)	35 (8.37%)	11737
Vaccine: Moderna	0 (0.00%)	0 (0.00%)	189 (8.14%)	0 (0.00%)	34 (8.13%)	11737
Vaccine: Pfizer	0 (0.00%)	0 (0.00%)	1739 (74.9%)	0 (0.00%)	319 (76.3%)	11737
**Symptoms**						
Allergy	0 (.%)	20 (95.2%)	134 (88.2%)	8 (100%)	17 (94.4%)	199
Diarrhea	0 (.%)	18 (94.7%)	133 (88.7%)	22 (100%)	28 (93.3%)	221
Drycough	0 (.%)	15 (93.8%)	94 (83.9%)	20 (100%)	51 (96.2%)	201
Fatigue	0 (.%)	9 (90.0%)	55 (75.3%)	17 (100%)	44 (95.7%)	146
Fever	0 (.%)	9 (90.0%)	96 (85.7%)	27 (100%)	51 (96.2%)	202
Headache	0 (.%)	53 (98.1%)	399 (97.1%)	33 (100%)	106 (100%)	604
Musclepain	0 (.%)	32 (97.0%)	265 (95.0%)	36 (100%)	87 (97.8%)	437
Nasalcongestion	0 (.%)	52 (96.3%)	391 (95.8%)	34 (97.1%)	83 (97.6%)	582
Sorethroat	0 (.%)	36 (97.3%)	186 (91.2%)	18 (100%)	56 (96.6%)	317
**Biochemical**						
Alkaline phosphatase (U/L)	73.4 (25.8)	-	-	-	-	221
Alanine transaminase (U/L)	22.7 (13.7)	18.6 (17.7)	21.8 (12.5)	22.0 (17.1)	21.8 (11.1)	11567
Aspartate transaminase (U/L)	37.3 (45.3)	-	-	-	-	531
Basophils(10^9/L)	0.04 (0.02)	0.04 (0.02)	0.04 (0.02)	0.04 (0.02)	0.03 (0.02)	11734
Bilirubin (mg/dL)	0.61 (0.38)	-	-	-	-	240
HDL Cholesterol (mg/dL)	60.6 (15.7)	67.3 (28.5)	62.4 (15.5)	65.2 (23.4)	63.9 (18.4)	11568
LDL Cholesterol (mg/dL)	113 (30.6)	106 (31.3)	116 (30.3)	110 (29.1)	113 (30.5)	10928
Cholesterol (mg/dL)	194 (34.3)	186 (35.1)	198 (34.2)	189 (34.0)	195 (35.1)	11568
Non-HDL Cholesterol (mg/dL)	133 (35.9)	123 (36.6)	136 (35.8)	126 (32.4)	132 (35.0)	11568
Mean corpuscular hemoglobin concentration (g/dL)	33.9 (0.87)	33.4 (1.00)	33.5 (1.05)	33.5 (1.12)	33.6 (1.07)	11734

	HC N=8664	HC1 N=238	HCV1 N=2322	NHR1 N=95	NHRV1 N=418	N
Mean corpuscular volume (fL)	90.1 (4.28)	90.4 (4.53)	90.4 (4.41)	90.3 (4.05)	90.1 (4.60)	11734
Creatinine (mg/dL)	0.87 (0.16)	0.83 (0.16)	0.86 (0.16)	0.86 (0.16)	0.87 (0.16)	11568
Eosinophils (10^9/L)	0.23 (0.16)	0.22 (0.17)	0.23 (0.16)	0.24 (0.16)	0.21 (0.15)	11734
Erythrocytes (10^9/L)	4.84 (0.42)	4.67 (0.43)	4.78 (0.42)	4.75 (0.36)	4.78 (0.41)	11734
ESR (mm/h)	7.97 (5.47)	7.71 (4.79)	7.56 (5.00)	7.33 (4.61)	7.51 (4.73)	11634
Fe (μg/dL)	72.0 (40.8)	-	-	-	-	173
Ferritin (ng/mL)	74.1 (110)	-	-	-	-	254
Fructosamine (μmol/L)	258 (48.7)	-	-	-	-	203
Gamma-glutamyltransferase (U/L)	22.5 (20.6)	15.9 (11.0)	21.3 (20.4)	19.1 (15.0)	19.9 (14.6)	11566
Glucose (mg/dL)	86.3 (12.4)	84.7 (13.5)	87.7 (14.5)	87.5 (23.6)	87.9 (11.7)	11568
Hematocrit (%)	43.5 (3.13)	42.1 (3.18)	43.1 (3.35)	42.8 (3.00)	42.9 (3.14)	11734
Hemoglobin (g/dL)	14.8 (1.21)	14.1 (1.23)	14.4 (1.28)	14.4 (1.23)	14.4 (1.20)	11734
Leukocytes (10^9/L)	6.73 (1.71)	6.81 (2.00)	6.67 (1.73)	6.49 (1.47)	6.46 (1.48)	11734
Lymphocytes (10^9/L)	2.34 (0.67)	2.31 (0.68)	2.31 (0.70)	2.14 (0.52)	2.29 (0.62)	11734
Monocytes (10^9/L)	0.62 (0.19)	0.61 (0.22)	0.64 (0.31)	0.57 (0.17)	0.62 (0.28)	11734
Neutrophils (10^9/L)	3.50 (1.26)	3.62 (1.57)	3.45 (1.39)	3.50 (1.23)	3.32 (1.19)	11734
Mean platelet volume (fL)	8.40 (0.69)	8.50 (0.70)	8.53 (0.73)	8.49 (0.68)	8.58 (0.71)	11733
Platelets (10^9/L)	238 (51.9)	245 (58.1)	234 (50.4)	239 (54.0)	232 (50.1)	11734
Red cell distribution (U)	13.3 (0.79)	13.4 (0.99)	13.5 (0.91)	13.4 (0.93)	13.4 (0.84)	11734
Transferrin (mg/dL)	283 (58.8)	-	-	-	-	172
Triglycerides (mg/dL)	98.4 (63.5)	84.0 (46.8)	96.7 (70.1)	87.5 (42.1)	95.7 (63.4)	11568
Urate (mg/dL)	5.13 (1.27)	4.80 (1.31)	5.18 (1.33)	5.26 (1.26)	5.33 (1.42)	11568

Table S4. Main performance metrics for COVID model. Cross-validation uses 10 repetitions of 5-fold; reported values are average and 95% confidence intervals (CI) for test subset. Permutation tests with 100 runs were used to estimate p-values.

Performance metric	Full model *value (p-value)*	Cross-validation *mean [95% CI] (p-value)*
AUC	0.998 (p <0.01)	0.998 [0.995, 0.999] (p <0.01)
Sensitivity	0.985 (p <0.01)	0.989 [0.970, 0.997] (p <0.01)
Specificity	0.983 (p <0.01)	0.983 [0.972, 0.994] (p <0.01)

Table S5. Main performance metrics for COVID lineage model. Cross-validation uses 10 repetitions of 5-fold; reported values are average and 95% confidence intervals (CI) for test subset. Permutation tests with 100 runs were used to estimate p-values.

Performance metric	Full model *value (p-value)*	Cross-validation *mean [95% CI] (p-value)*
AUC	0.922 (p <0.01)	0.907 [0.821, 0.971] (p <0.01)
Sensitivity	0.887 (p = 0.01)	0.908 [0.783, 1.000] (p <0.01)
Specificity	0.861 (p <0.01)	0.847 [0.662, 0.990] (p <0.01)

Table S6. Main performance metrics for age-gender balanced COVID model. Cross-validation uses 10 repetitions of 5-fold; reported values are average and 95% confidence intervals (CI) for test subset. Permutation tests with 100 runs were used to estimate p-values.

Performance metric	Full model *value (p-value)*	Cross-validation *mean [95% CI] (p-value)*
AUC	0.997 (p <0.01)	0.996 [0.990, 1.000] (p <0.01)
Sensitivity	0.981 (p <0.01)	0.986 [0.959, 1.000] (p <0.01)
Specificity	0.980 (p <0.01)	0.980 [0.950, 1.000] (p <0.01)

Figure S1. Principal Component Analysis (PCA) score plot showing the first and second components that explains the maximum variability (19.7% and 15.1% respectively) of metabolomics data from acute patients at different hospitals. Ellipses represent the 95% confidence intervals.

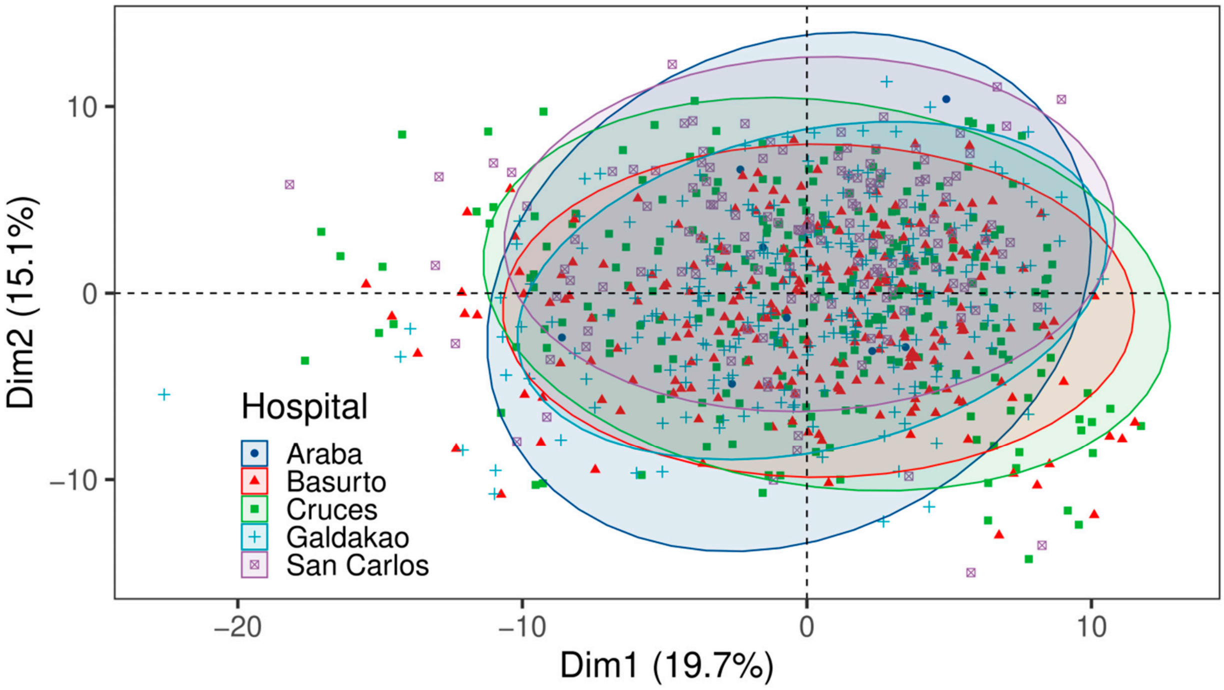

Figure S2. Concentration of chemokines and cytokines that are already associated to the COVID-19 immunological response in different time periods from disease onset (in days) and compared to healthy cohort (HC). Statistically significant comparisons (unadjusted p-value < 0.05) versus HC group are marked with asterisks (*: p-value < 0.05; **: p-value < 0.01; ***: p-value < 0.001; ****: p-value < 0.0001).

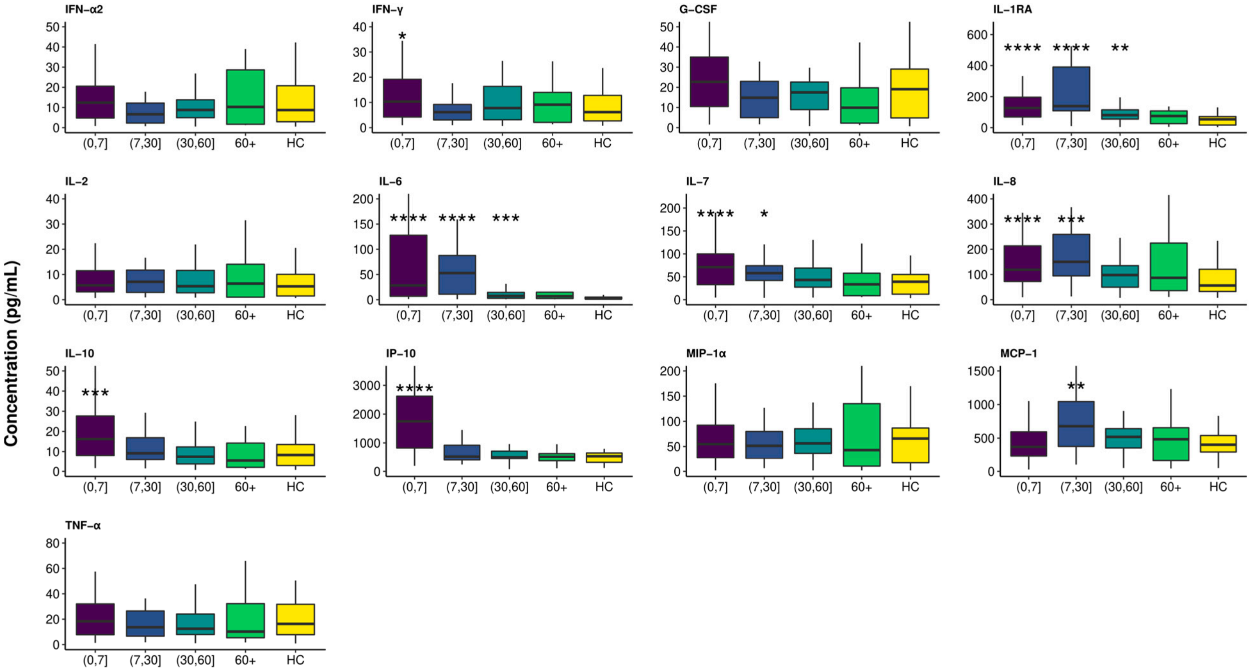

Figure S3. O-PLS-DA score plot from COVID model with the projection of non-hospitalized recovered (NHR) people (triangles). Those NHR people whose disease onset is less than 1 month are represented in orange.

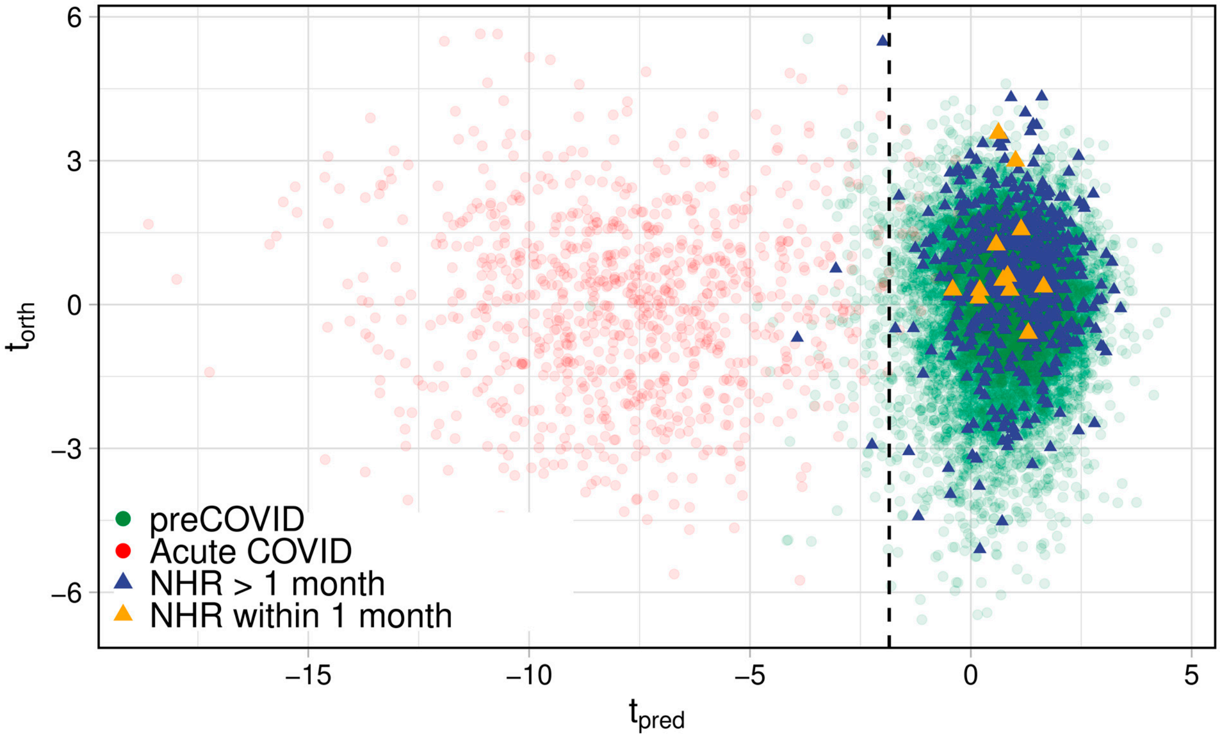

Figure S4. Comparison of inflammation markers, cytokine and chemokine levels of non-hospitalized recovered (NHR) group within 1.5 months from COVID onset versus controls (HC). The horizontal position of each point represents the size effect in the comparison as the binary logarithm of fold-change (mean in NHR group divided by mean of control group); its horizontal line is the associated standard error. If the difference is statistically significant (unadjusted p-value < 0.05) the point is filled.

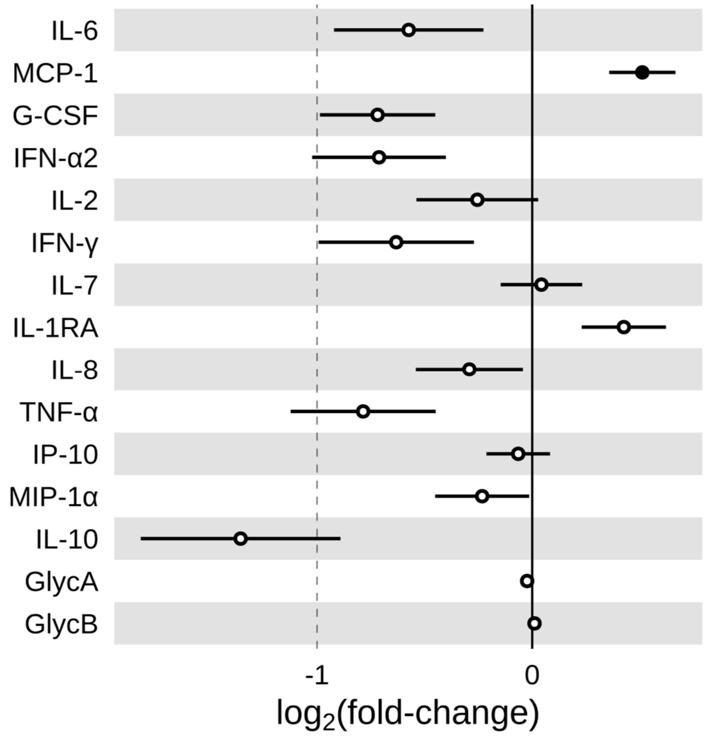

Figure S5. Concentration evolution for the 38 metabolomic parameters used to build the COVID model. Evolution goes from acute phase (AC) through the different recovery periods (RE). Non-hospitalized recovered (NHR) people are included at the end as reference. Horizontal dashed line shows the mean concentration in control group (HC). Each point represents the mean value, standard errors are included as vertical lines. Concentration units: mmol/L (2-Hydroxybutyric acid, 2-Oxoglutaric acid, Acetoacetic acid, Acetone, Choline, Citric acid, Creatine, Formic acid, Glucose, Glutamic acid, Glutamine, Histidine, Lactic acid, Lysine, Phenylalanine, Pyruvic acid), mg/dL (HDL-1 Free Cholesterol, HDL-2 Triglycerides, HDL-3 Apo-A2, HDL-3 Free Cholesterol, HDL-4 Apo-A1, HDL-4 Triglycerides, LDL Triglycerides, LDL-1 Cholesterol, LDL-2 Triglycerides, LDL-3 Cholesterol, LDL-4 Cholesterol, LDL-5 Free Cholesterol, LDL-6 Triglycerides, Total Cholesterol, VLDL-4 Triglycerides, VLDL-5 Free Cholesterol) and arbitrary units (ratios, GlycA and GlycB).

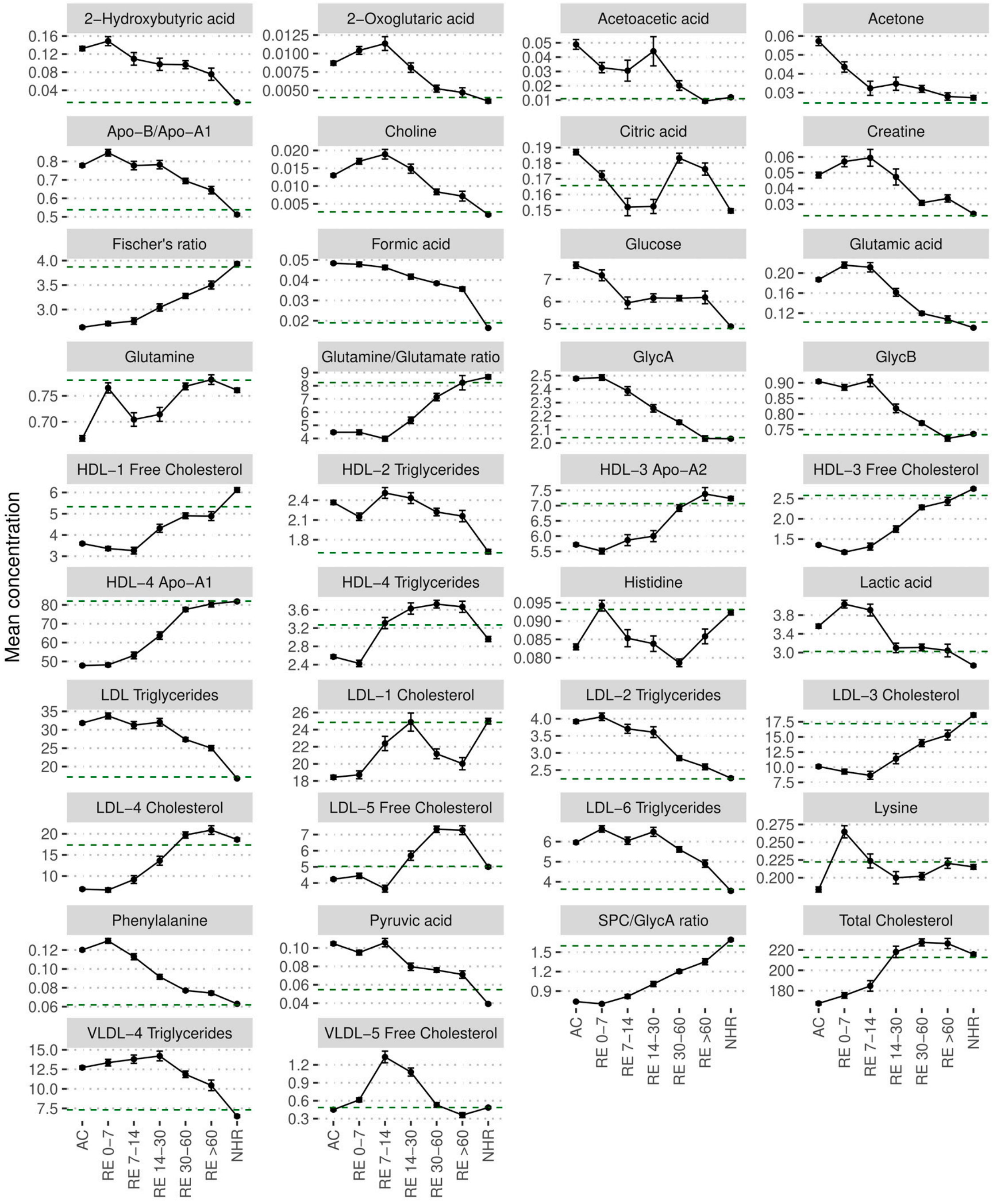

Figure S6. Comparison of acute COVID and the different recovery stages versus the control group (HC) for each analyzed metabolic parameter. The horizontal position of each point represents the size effect in the comparison in standard deviation (SD) units; its horizontal line is the associated standard error. If the difference is statistically significant (p-value < 0.05 after FDR correction) the point is filled.

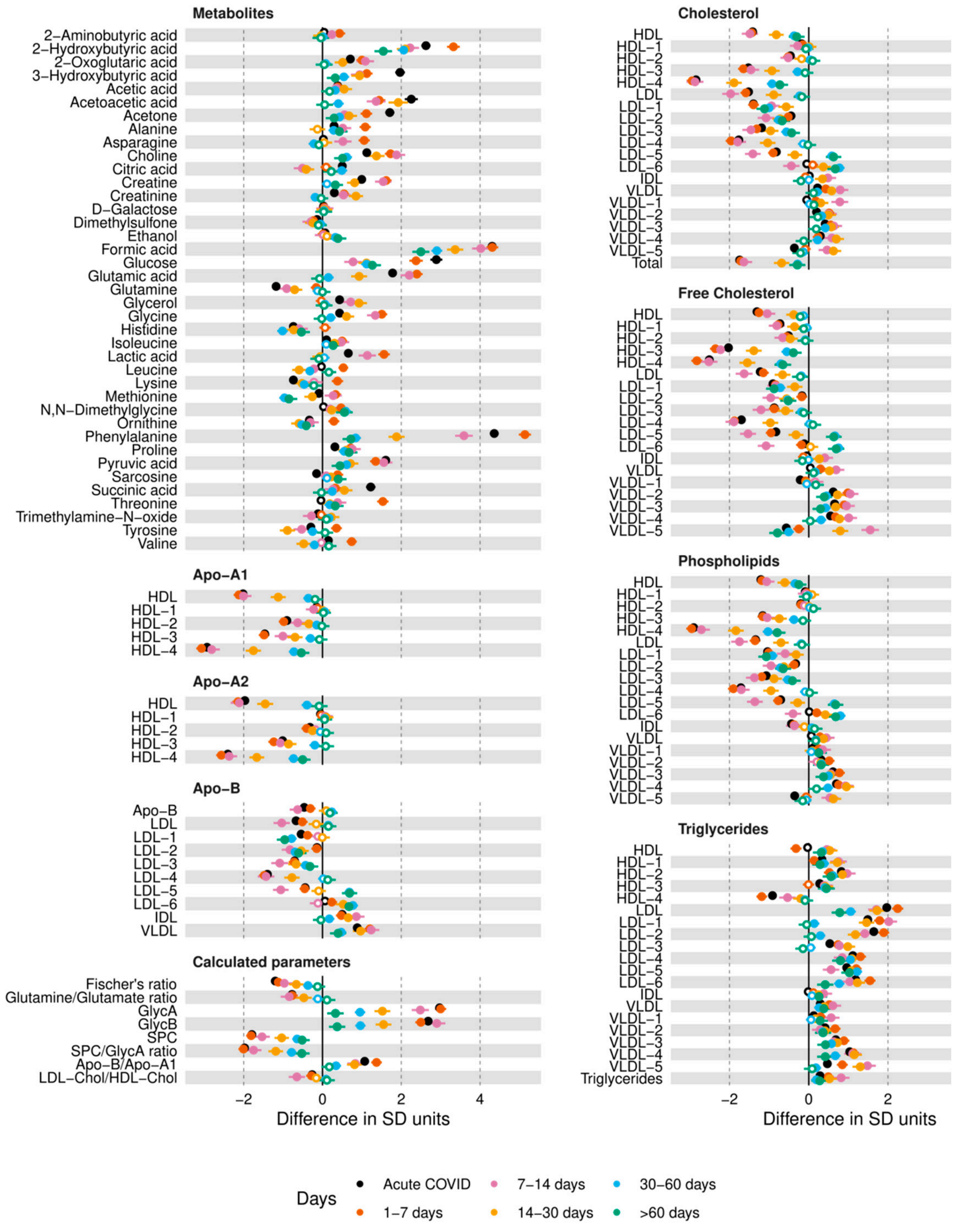

Figure S7. Effect of vaccination between HCV1 and HC1 cohorts for each analyzed metabolic parameter. The horizontal position of each point represents the size effect in the comparison in standard deviation (SD) units; its horizontal line is the associated standard error. If the difference is statistically significant (p-value < 0.05 after FDR correction) the point is filled.

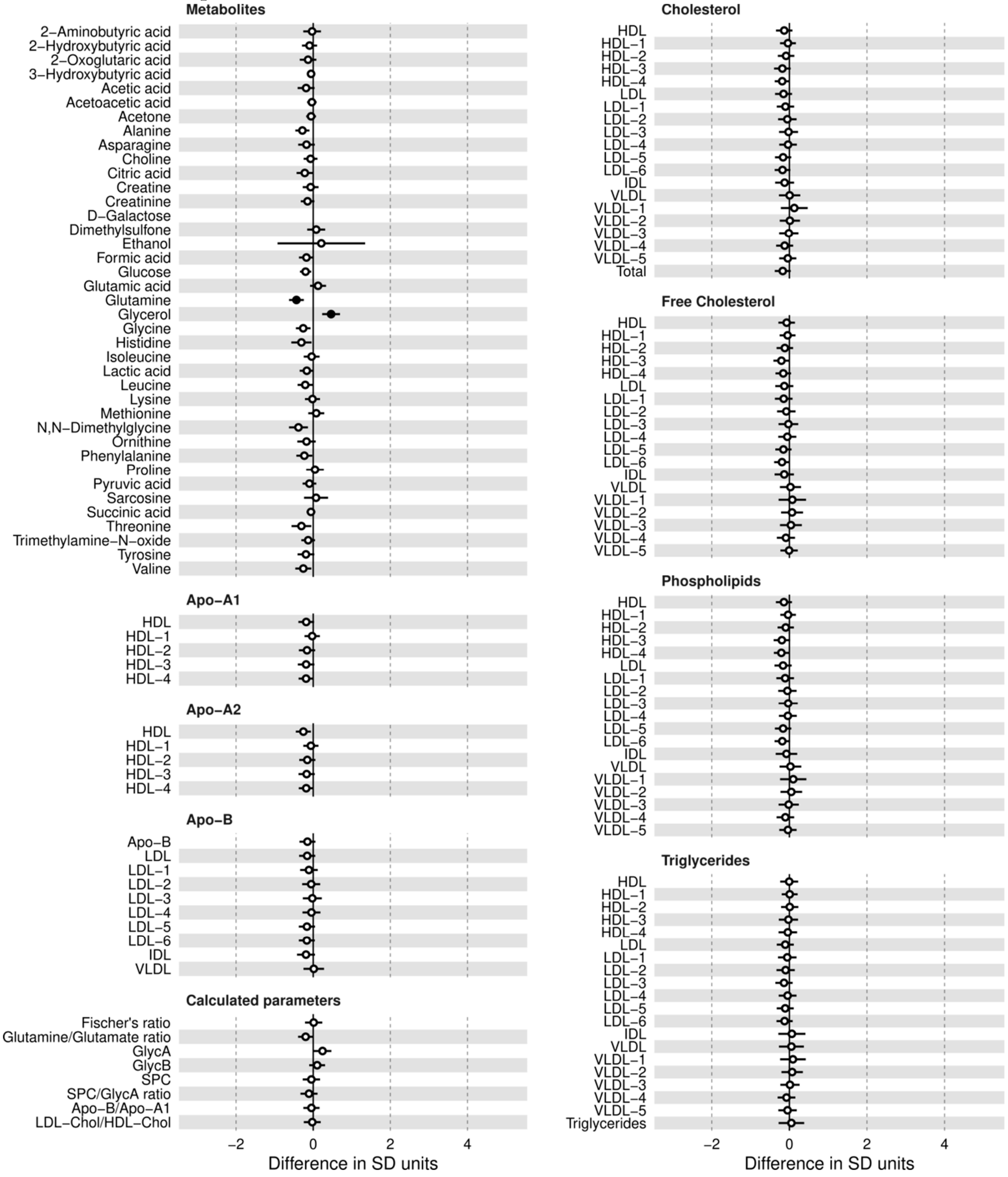

Figure S8. Violin plots showing the effect of vaccines by type for the concentration of Glutamine and Glycerol compared to people without vaccination (none). Horizontal bars indicate statistical significance according to p-value from Student's t tests. ns: non-significant; *: p-value < 0.05; **: p-value < 0.01

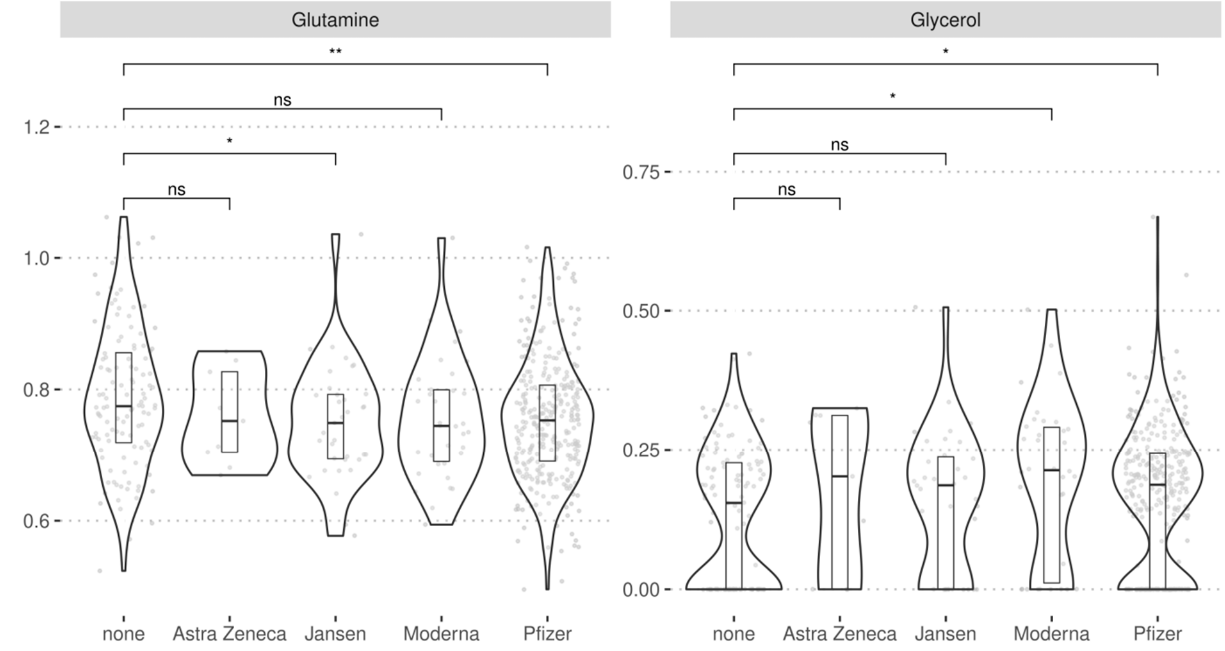

Figure S9. COVID model using a subset of acute COVID (AC) and control cohorts (HC) that have been matched between them by gender and age. A) Score plot from O-PLS-DA model showing the discrimination between COVID and controls. The vertical dashed line indicates the threshold that maximized the Youden's index in receiver operating characteristic (ROC) analysis. B) ROC curve for model shown in figure S9A. The area under the curve (AUC) is 0.997. In red it is indicated the t_{pred} value that maximizes the Youden's index, and the resulting specificity and sensitivity between parenthesis. C) Distribution of gender and number of samples by subcohort. D) Distribution of ages by gender and subcohort.

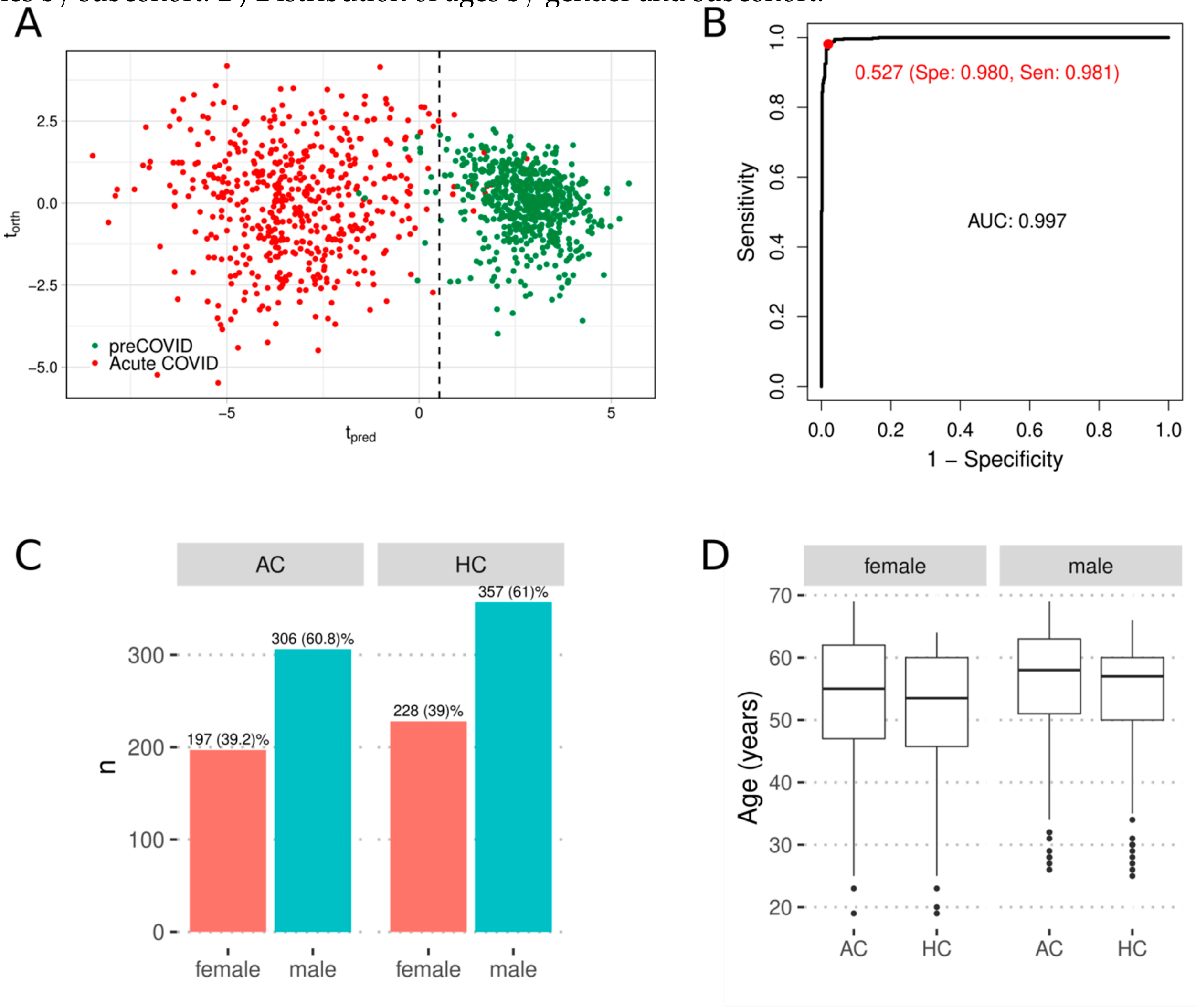

Figure S10. Comparison of O-PLS-DA loadings from predictive components of two COVID models by gender: one using only women and other using only men.

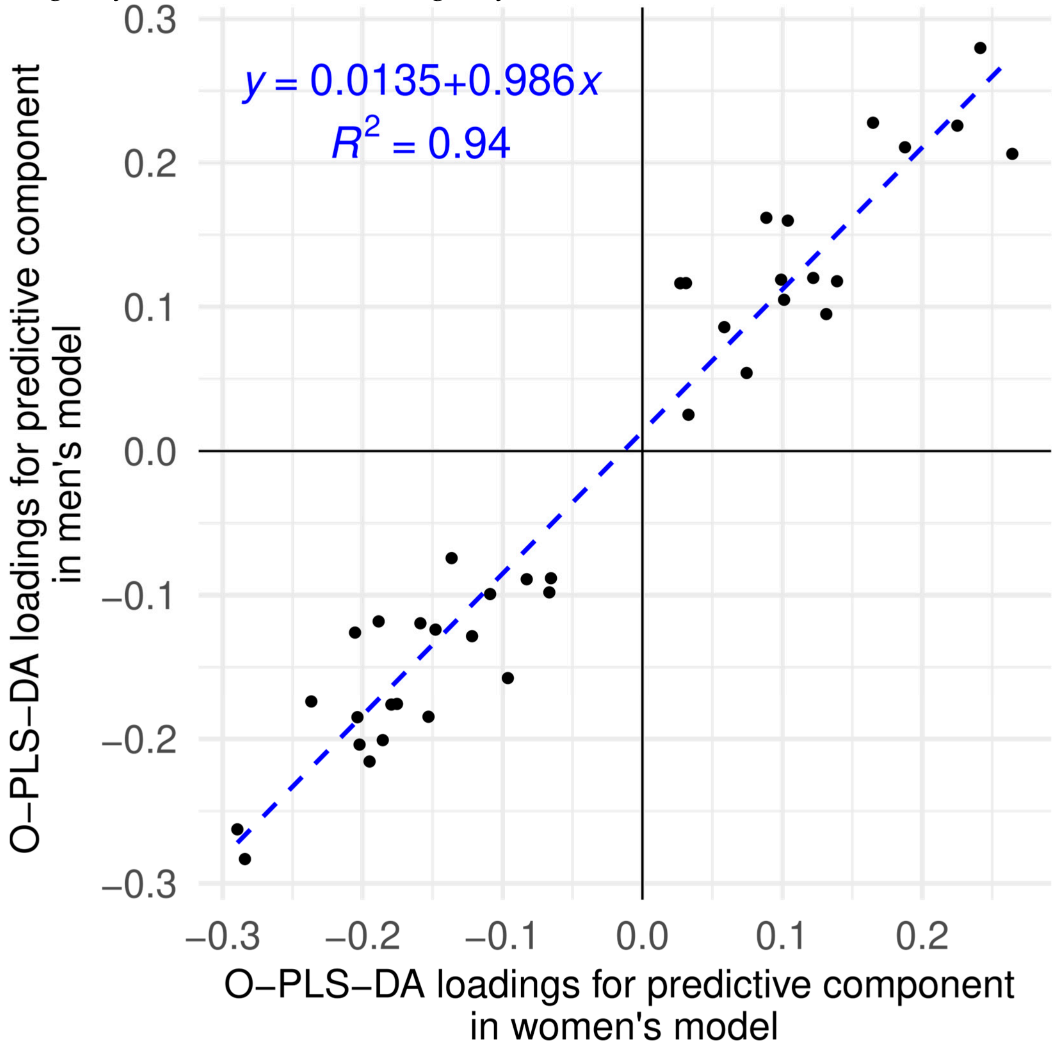

Figure S11. Estimation of the effect of medication in COVID acute patients after 1-2 days at hospital as compared versus initial collected sample at hospitalization. The horizontal position of each point represents the size effect in the comparison in standard deviation (SD) units; its horizontal line is the associated standard error. If the difference is statistically significant (p-value < 0.05 after FDR correction) the point is filled.

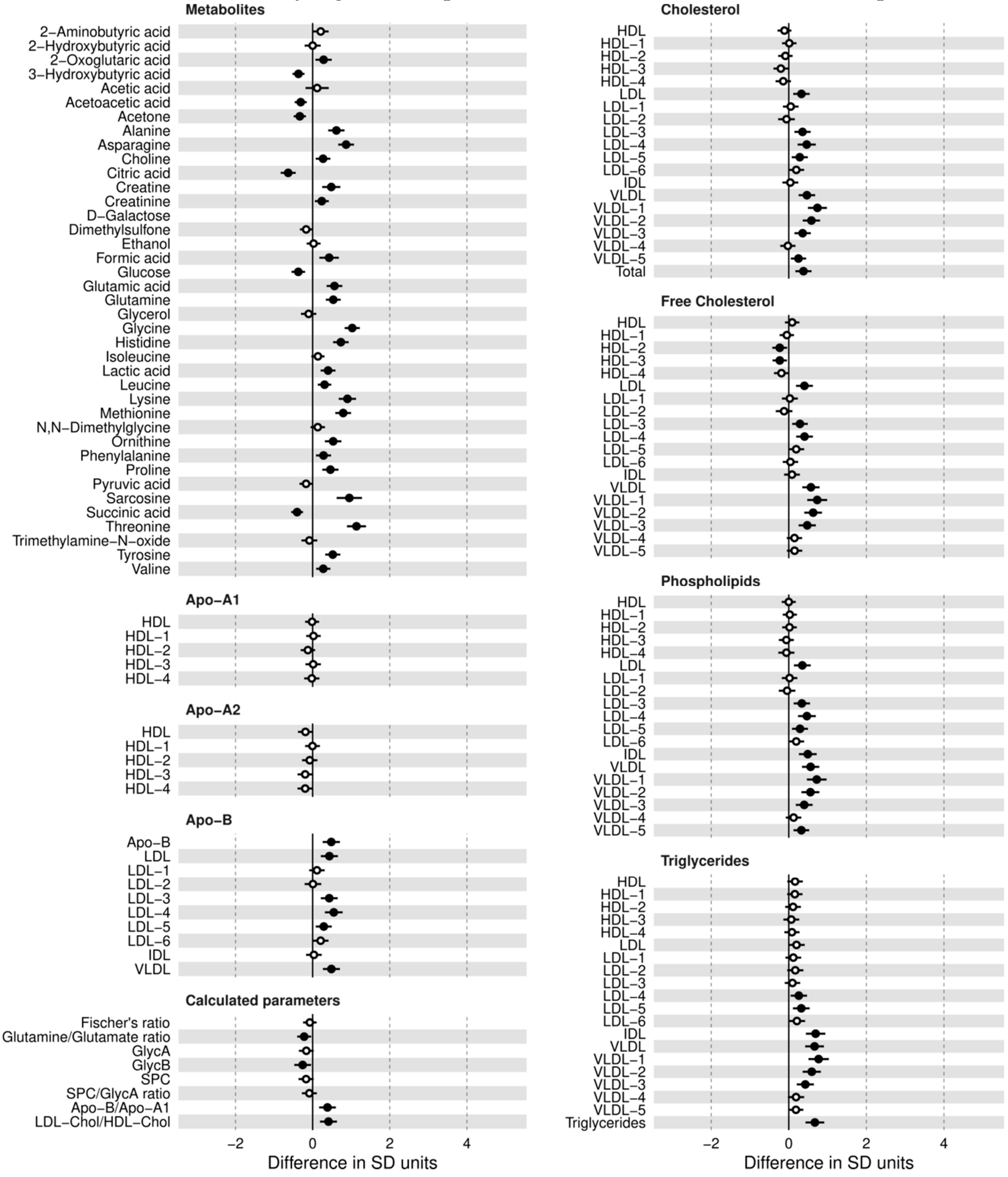

Figure S12. Estimation of the effect of medication on cytokine and chemokine levels for COVID acute patients after 1-2 days at hospital as compared versus initial collected sample at hospitalization. The horizontal position of each point represents the size effect in the comparison as the binary logarithm of fold-change (mean in mild group divided by mean of control group); its horizontal line is the associated standard error. If the difference is statistically significant (unadjusted p-value < 0.05) the point is filled.

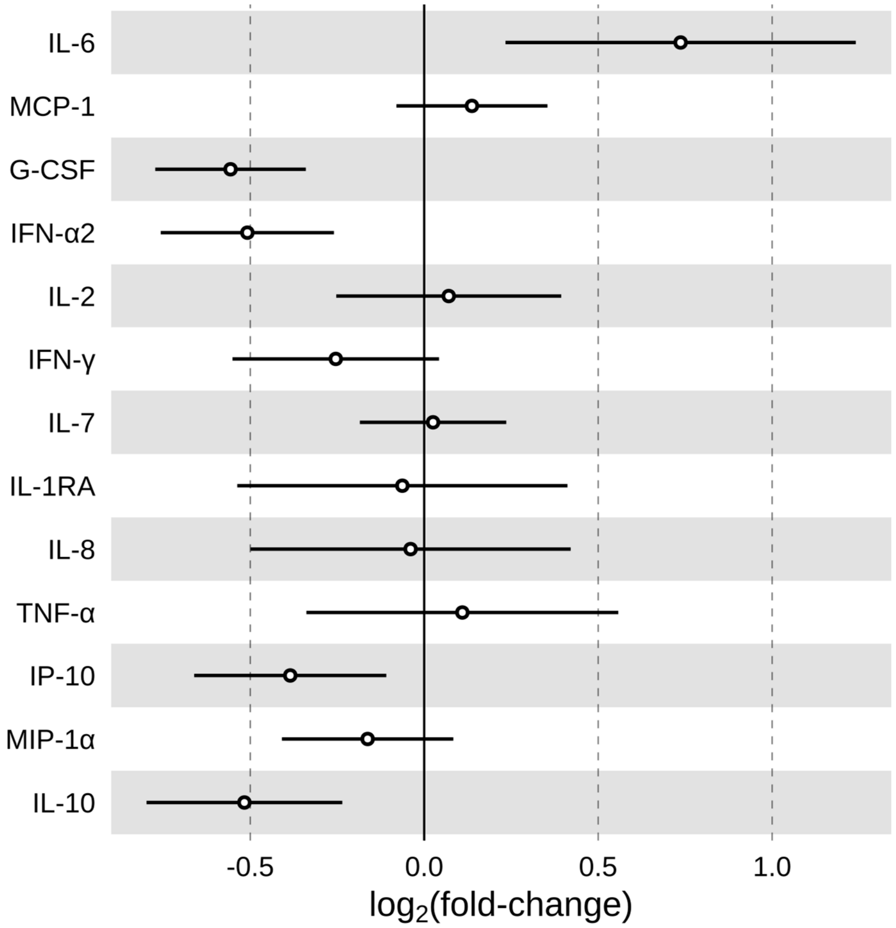

Supplement: Supplementary file 1 [file metabolites-12-01206-s001.zip › metabolites-2044738-supplementary.pdf]
